# Integrated dose–response metabolomics with therapeutic effects and adverse reactions may demystify the dosage of traditional Chinese medicine

**DOI:** 10.1186/s13020-022-00687-4

**Published:** 2022-11-19

**Authors:** Yan-Yan Chen, Jia-Qian Chen, Yu-Ping Tang, Er-Xin Shang, Qi Zhao, Jun-Bo Zou, Ding-Qiao Xu, Shi-Jun Yue, Jie Yang, Rui-Jia Fu, Gui-Sheng Zhou, Jin-Ao Duan

**Affiliations:** 1 Key Laboratory of Shaanxi Administration of Traditional Chinese Medicine for TCM Compatibility, and State Key Laboratory of Research and Development of Characteristic Qin Medicine Resources (Cultivation), and Shaanxi Key Laboratory of Chinese Medicine Fundamentals and New Drugs Research, and Shaanxi Collaborative Innovation Center of Chinese Medicinal Resources Industrialization, Shaanxi University of Chinese Medicine, Xi’an, 712046 Shaanxi Province China; 2 Jiangsu Collaborative Innovation Center of Chinese Medicinal Resources Industrialization, and National and Local Collaborative Engineering Center of Chinese Medicinal Resources Industrialization and Formulae Innovative Medicine, and Jiangsu Key Laboratory for High Technology Research of TCM Formulae, Nanjing University of Chinese Medicine, Nanjing, 210023 Jiangsu Province China

**Keywords:** Dose–response, Metabolomics, Therapeutic effect, Adverse reaction, Traditional Chinese medicine, Rhubarb

## Abstract

**Background:**

Traditional Chinese medicine (TCM) has been used to treat various diseases for thousands of years. However, the uncertainty of dosage as well as the lack of systemic evaluation of pharmacology and toxicology is one major reason why TCM remains mysterious and is not accepted worldwide. Hence, we aimed to propose an integrated dose–response metabolomics strategy based on both therapeutic effects and adverse reactions to guide the TCM dosage in treatment.

**Methods:**

The proposed methodology of integrated dose–response metabolomics includes four steps: dose design, multiple comparison of metabolic features, response calculation and dose–response curve fitting. By comparing the changes of all metabolites under different doses and calculating these changes through superposition, it is possible to characterize the global disturbance and thus describe the overall effect and toxicity of TCM induced by different doses. Rhubarb, commonly used for constipation treatment, was selected as a representative TCM.

**Results:**

This developed strategy was successfully applied to rhubarb. The dose–response curves clearly showed the efficacy and adverse reactions of rhubarb at different doses. The rhubarb dose of 0.69 g/kg (corresponding to 7.66 g in clinic) was selected as the optimal dose because it was 90% of the effective dose and three adverse reactions were acceptable in this case.

**Conclusion:**

An integrated dose–response metabolomics strategy reflecting both therapeutic effects and adverse reactions was established for the first time, which we believe is helpful to uncover the mysterious veil of TCM dosage. In addition, this strategy benefits the modernization and internationalization of TCM, and broadens the application of metabolomics.

**Supplementary Information:**

The online version contains supplementary material available at 10.1186/s13020-022-00687-4.

## Introduction

Traditional Chinese medicine (TCM) has been practiced in China for thousands of years and has played an important role in fighting diseases and maintaining health. This ancient Chinese wisdom has been accumulated through thousands of trial-and-error practices in the long struggle against various diseases [1]. However, TCM has long been criticized as being deficient in scientific evidence due to its empirical basis. Therefore, the previous “experiential, extensive and mysterious” development mode must be changed, in which the dosage of TCM has always been the focus of controversy. Demystifying the dosage of TCM will help to eliminate the doubts over it.

TCM has characteristics of efficacy and toxicity, and “Water can float, but also can capsize” is a good summary of the dual role. TCM is often erroneously believed to be non-toxic or low-toxic because it is traditionally considered as “natural”. However, the toxicity and side effects of TCM should not be ignored. Whether TCM is a poison or a remedy depends largely on the dosage we use. How much difference is there between the dose that distinguishes a remedy from a poison? Dose–response relationship quantitatively defines the role of drug dose in inducing a biological response [2]. Nowadays, the research mode of dose–response relationship of chemical medicine has been gradually improved, since the active components and the targets are usually clear. However, the relationship between dosage and response of TCM is still in the exploratory stage. The multi-component, multi-target, and multi-efficacy characteristics of TCM bring difficulties in evaluating the dose–response relationship of TCM.

In the past, TCM dosage was usually derived from doctor’s clinical experience, which could be subjective and often prompts uncertainty. Currently, some progress has been made in the dose–response relationship of TCM: (1) Based on data mining technology, that is, establishing TCM application database on the basis of ancient and modern literatures, and using computer data mining (such as association rule analysis, artificial neural network, etc.) to explore the change pattern between dose and response according to clinical efficacy and drug characteristics [3]; (2) Based on the experimental research, of which the most commonly used are traditional pharmacological and toxicological studies. This research mode can roughly identify the trends of dose–response relationship through different dosage groups (mostly designed as high, medium and low dose), and is relatively simple to implement. However, the selection of evaluating indicators is the key to the final outcome. Different indicators often exhibit different dose–response curves, and sometimes different trends of curves can be confusing. Although the multi index comprehensive method has also been developed for overall evaluation, the result is not always satisfactory.

Metabolomics is a systemic approach to study the complete set of metabolites in biological system and provides a top-down and holistic perspective of the global metabolic state, which is coincide with the holistic nature of TCM. Thus, it has been widely applied in many research fields of TCM. By selecting and integrating nine biomarkers produced by untargeted metabolomics, the dose–response characteristics of rhubarb in cholestasis model was revealed [4]. Through nonlinearly fitting the relative distance of the model group and treated group based on metabolomics, the dose–effect curve of rhubarb for treating constipation was constructed [5]. A previous study introduced a dose–response metabolomics workflow to understand biochemical mechanisms of small molecule drugs [6]. However, these studies are still flawed in systematically clarifying the dosage-response relationship. Two major limitations remain to be addressed: firstly, it is difficult to fully reflect drug efficacy or toxicity by selecting only a few specific metabolites; secondly, the effect and toxicity of drugs are often accompanied, and focusing on only one aspect is not sufficient.

Since the efficacy and toxicity of TCM are accompanied by each other, under appropriate dose, TCM mainly plays a role in treating diseases, but when the dose exceeds a certain range, adverse reactions may occur. When the adverse reactions are mild, the balance of endogenous metabolites is disturbed, but it’s not enough to cause organic changes, so it is difficult to observe the adverse reactions by traditional pharmacological research; only when the level of endogenous metabolites changes from quantitative to qualitative and appropriate toxicity index is selected, the toxic reaction can be obviously observed. In contrast to classical biochemical approaches that focus on single indicator, metabolomics involves the collection of quantitative data on a broad series of metabolites to gain an overall understanding of metabolism associated with drug exposure [7]. Metabolomics provides quantitative comparisons of metabolite concentrations between samples, which offers unique advantages over traditional pharmacological research. Metabolite concentrations provide a direct readout of biochemical activity, and changes in metabolite levels signifies alterations in phenotype [8]. Toward this end, we propose an integrated dose–response metabolomics methodology based on both therapeutic effects and adverse reactions to guide dosing in TCM treatment. By comparing the changes of all metabolites under different doses and calculating these changes through superposition, global disturbances could be characterized. Since most biological processes depend upon metabolism, it provides an opportunity to better define these relationships between biological functions and biochemical pathways, some of which are likely to be unexpected, so as to characterize the overall effect and toxicity of TCM caused by different doses.

## Materials and methods

### Plant materials and quality control

The dried roots and rhizomes of *Rheum tanguticum* Maxim. ex Balf. (No. SUCM-20190106) were collected from Gannan Autonomous Prefecture, Gansu Province, China. They were identified as the four-year-old authentic rhubarb by Professor YongGang Yan (Shaanxi University of Chinese Medicine, Xi’an, China), and the voucher specimens were deposited in the Herbarium of Shaanxi University of Chinese Medicine. Quality control of rhubarb can be found in Additional file 1.

### Animal experiments

Male Sprague Dawley rats were randomly divided into eight groups: normal group, model group, and six doses of rhubarb groups (*n* = 8). The constipation model was established to observe the effect of rhubarb. The defecation characteristics and pathological sections of colons were employed for purgative effect and toxicity evaluation. Serum samples were collected for metabolomics analysis. The animal experiments were approved by the Animals Ethics Committee of Shaanxi University of Chinese Medicine (No. 2020078). Detailed experimental design and procedures can be found in Additional file 1.

### Data acquisition

The metabolic profiling of serum samples was performed by an Acquity™ UPLC system (Waters, Milford, USA) coupled to a Synapt™ Q-TOF mass spectrometer. Raw UPLC-Q-TOF/MS data were processed by MassLynx^TM^v4.1 software (Waters, Milford, MA, USA) for peak detection, noise removal, filtering and alignment to generate a data matrix that was composed of retention time, *m/z* value and normalized ion intensity for each peak area.

### Dose–response modeling

**Fig. 1:**
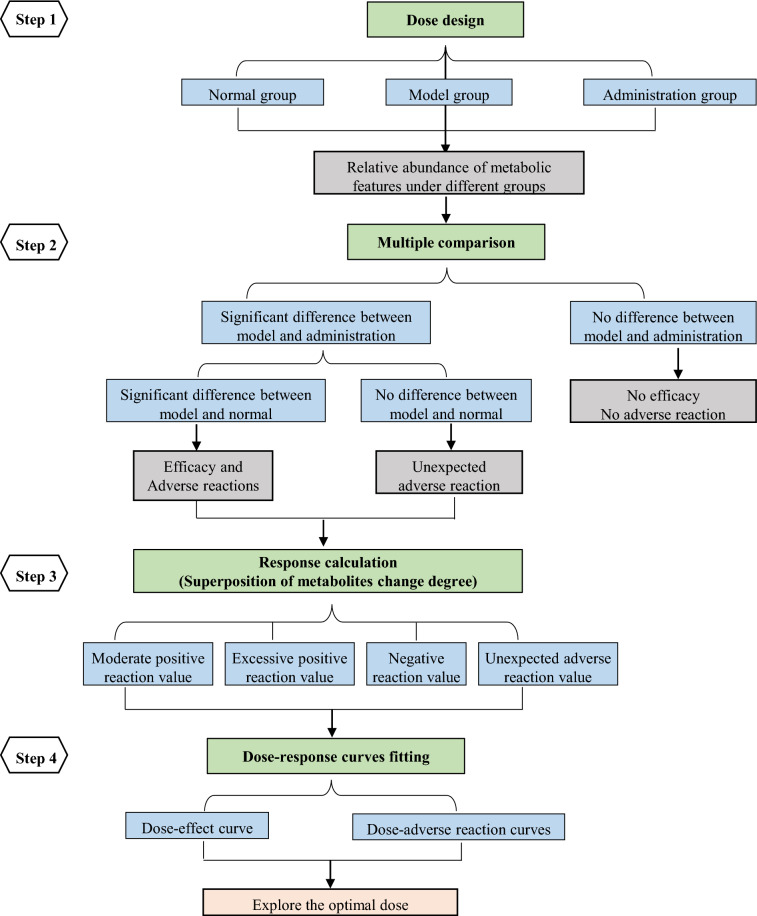
Overall workflow for dose–response metabolomics with therapeutic effects and adverse reactions

Dose–response modeling mainly consists of four steps: dose design, multiple comparison between groups, degree value calculation of the changed features, and fitting of dose–response curves. The overall workflow for dose–response metabolomics with therapeutic effects and adverse reactions was shown in Fig. 1.

**Fig. 2:**
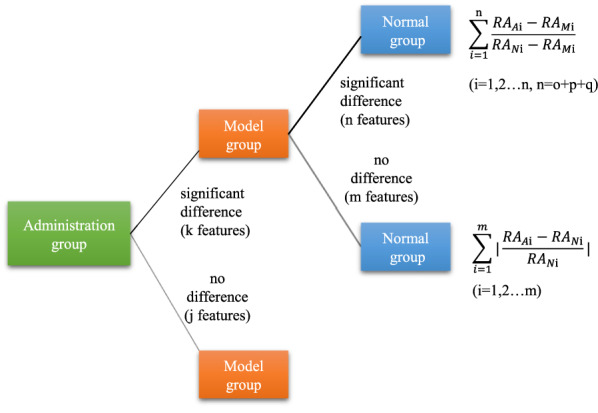
Multiple comparison of metabolic features between groups

For multiple comparisons, one way analysis of variance is performed to compare the relative abundance (RA) of each detected feature. If there are no significant differences (*P* < 0.05 or *P* < 0.01, user defined *P* value) between the administration group and the model group, the features are considered meaningless. If significant differences exist between the model group and the normal group, as well as the administration group and the model group, it can be divided into three situations according to the regulation degree of TCM: “moderate positive reaction”, “excessive positive reaction” and “negative reaction”. If there are no difference between the model group and the normal group, while significant differences exist between the administration group and the normal group/the model group, it means that the modeling did not cause change in metabolites but the administration caused metabolites disturbance, which are assigned as “unexpected reaction” induced by TCM. Multiple comparisons between the groups and the formula are shown in Fig. 2.

Then, degree value of the changed features is calculated by applying Eqs. (1)–(6). Degree_*i*_ in Eq. (1) has a common premise that TCM can adjust the metabolites disorder caused by modeling, and there are three situations: when 0 < Degree_*i*_ ≤ 1, it indicates that TCM can adjust the level of metabolites to near normal or normal, which is defined as “moderate positive reaction” and is the so-called “efficacy”, in addition, the “moderate positive reaction” value is expressed as the sum of the deviation degree and can be calculated by Eq. (2); when Degree_*i*_ > 1, it means that TCM overregulates the metabolites to higher than normal levels, which is assigned as “excessive positive reaction”, whose value is expressed as the sum of the deviation degree and can be calculated by Eq. (3); when Degree_*i*_ < 0, it shows that TCM not only fails to adjust the level of metabolites to the normal level, but also can regulate them to the opposite direction, which is defined as “negative reaction” and is also a type of adverse reactions, whose value is expressed as the sum of the deviation degree and can be calculated by Eq. (4). When Degree_*i*_ in Eq. (5) is less than 0, it indicates that TCM administration causes the down-regulation of metabolites, at the same time, when Degree_*i*_ in Eq. (5) is more than 0, it shows the up-regulation of metabolites, both of which mean that TCM can lead to the metabolites to deviate from the normal level. It is defined as “unexpected reaction” and is expressed as the sum of the absolute value of the deviation degree, which can be calculated by Eq. (6).

1 $${\text{Degree}}_i = \frac{{RA_{Ai} - RA_{Mi} }}{{RA_{Ni} - RA_{Mi} }}, \, \left( {i = 1,2 \ldots n} \right)$$

2 $${\text{If}}\,\,\,0 < \frac{{RA_{Ai} - RA_{Mi} }}{{RA_{Ni} - RA_{Mi} }} \le 1,\,{\text{Value}}\,\left( {\text{moderate positive reaction}} \right) = \mathop \sum \limits_{i = 1}^q \frac{{RA_{Ai} - RA_{Mi} }}{{RA_{Ni} - RA_{Mi} }},\,\left( {i = 1,2 \ldots q} \right)$$

3 $${\text{If}}\,\,\frac{{RA_{Ai} - RA_{Mi} }}{{RA_{Ni} - RA_{Mi} }} > 1,\,{\text{Value}}\,\left( {{\text{excessive}}\,{\text{positive}}\,{\text{reaction}}} \right) = \mathop \sum \limits_{i = 1}^p \frac{{RA_{Ai} - RA_{Mi} }}{{RA_{Ni} - RA_{Mi} }}, \, \left( {i = 1,2 \ldots p} \right)$$

4 $${\text{If}}\,\frac{{RA_{Ai} - RA_{Mi} }}{{RA_{Ni} - RA_{Mi} }} < 0,\,{\text{Value}}\,\left( {{\text{negative}}\,{\text{reaction}}} \right) = \mathop \sum \limits_{i = 1}^0 \frac{{RA_{Ai} - RA_{Mi} }}{{RA_{Ni} - RA_{Mi} }},\left( {i = 1,2 \ldots 0} \right)$$

5 $${\text{Degree}}_i = \frac{{RA_{Ai} - RA_{Ni} }}{{RA_{Ni} }},\left( {i = 1,2 \ldots m} \right)$$

6 $${\text{Value}}\,\left( {\text{unexpected reaction}} \right)\, = \mathop \sum \limits_{i = 1}^m \left| {\frac{{RA_{Ai} - RA_{Ni} }}{{RA_{Ni} }}} \right|,\left( {i = 1,2 \ldots m} \right)$$

RA_*Ai*_ means the relative abundance of the feature in administration group, and RA_*Ni*_ means the relative abundance of the feature in normal group, then RA_*Mi*_ means the relative abundance of the feature in model group.

The final step is to fit the dose–response curves, and dose–response models are regression models where the independent variable (x) refers to the dose and the dependent variable (y) refers to the response (efficacy or adverse reactions), in which y is the value derived from the metabolomics features according to the Eqs. (2), (3), (4) and (6). The positive value of y indicates the efficacy, and the negative value of y represents the adverse reactions. The basic Trendline package in R. Plot was used to construct the dose–response curves, the regression lines and confidence intervals were drawn after, and the models built in the ‘trendline’ function were used to display the regression equations, R-squares and P-values. The following R code was run:

$$> {\text{ library }}\left( {{\text{basicTrendline}}} \right)$$

$$> {\text{ trendline }}\left( {{\text{x}},{\text{ y}},{\text{ model}} = {^{\prime\prime}}{\text{exp3P}}{^{\prime\prime}},{\text{ CI}}.{\text{fill}} = {\text{FALSE}},{\text{ summary}} = {\text{TRUE}},{\text{ eDigit}} = {4}} \right)$$

“exp3P” indicates the formula of y = a×${e}^{\mathrm{bx}}$ + c; level of confidence interval was 0.95 by default; R^2^ indicates the R-Squared value of regression model; *p* indicates the *p*-value of regression model; AIC or BIC indicates the Akaike’s information criterion or Bayesian information criterion for fitted model, the smaller the AIC or BIC, the better the model; RSS indicates the residual sum of squares of regression model.

## Results

### Multicomponent quantification of rhubarb

Fifteen components of rhubarb were quantified. The Calibration curves of 15 analytes were showed in Additional file 1: Table S1. The representative HPLC chromatograms of the mixed standards and the rhubarb decoction were showed in Additional file 1: Fig. S1. The total content of aloe emodin, rhein, emodin, chrysophanol, and physcion was 0.273%, which was in accordance with the quality standard of Chinese Pharmacopoeia (2020).

### Effect of rhubarb on constipation model

The results showed that high-calorie diet significantly reduced the fecal pellets number, fecal pellets weight and fecal water content (Additional file 1: Fig. S2), which implies the construction of a constipation model. The evacuation index and fecal water content increased in all groups received rhubarb, with the most pronounced changes in the high dose groups (Rhubarb 3–Rhubarb 6) (Additional file 1: Fig. S3). The highest dose groups (Rhubarb 5–Rhubarb 6) existed exfoliated intestinal epithelial tissues and inflammatory infiltration (Additional file 1: Fig. S4).

### Metabolic profiles and multiple comparisons of metabolic features

**Fig. 3:**
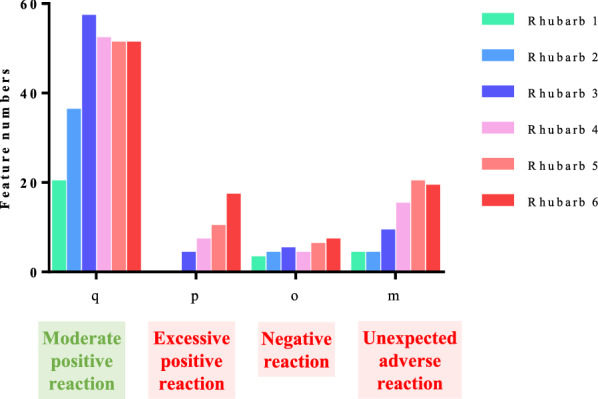
Feature numbers changed with the rhubarb dose (q, p, o, m represents feature numbers of moderate positive reaction, excessive positive reaction, negative reaction, and unexpected adverse reaction)

Our approach relies on comparing the metabolic features of the normal group, the model group and the administration group receiving various doses. Untargeted metabolomics based on UPLC-Q-TOF/MS was employed here, which holds great potential in conferring comprehensive snapshots of metabolic phenotypes by profiling small molecules in multiple matrices. In serum samples, 462 metabolic features were detected totally (Additional file 1: Table S2), 100 of which showed significant differences (*P* < 0.05) in RA between the model and rhubarb groups. Further comparisons between the model and normal groups showed that 75 of the 100 features have significant differences. According to the regulation ability of rhubarb to the metabolites changed by modeling, there were three situations: according to Eq. (1), when 0 < Degree_*i*_ ≤ 1, it means that rhubarb can adjust the disturbance metabolites to near normal or normal level, and feature numbers increased from 20 to 57 and then reduced to 51 with the dosage change, which is “moderate positive reaction” and represents the so-called “efficacy”; when Degree_*i*_ is more than 1, it means that rhubarb overregulates the metabolites level, and feature numbers changed from 0 to 17 as the dose increased, which is “excessive positive reaction”; when Degree_*i*_ is less than 0, it means that rhubarb regulates the metabolites in the opposite direction compared with the normal group and is “negative reaction”, and feature numbers increased with the dose from 3 to 7, indicating that high dose of rhubarb treatment would augment the adverse reaction; 25 out of the 100 features have no significant difference between the model and normal groups, which indicated that rhubarb, rather than the model, could cause the unexpected metabolic imbalance, and the disturbance increased with the dose, suggesting that excessive dose of rhubarb may induce unexpected reaction (the feature numbers in Fig. 3). The results showed that the higher the dose, the more obvious the adverse reactions, but the efficacy dose not increase infinitely.

### Response calculation

To describe the outcomes reflecting efficacy and adverse reactions, the deviation degree of significant changed features in each situation were superimposed, which is more exact and comprehensive than using a single variable. As shown in Table 1, three types of adverse reactions were all increased while the efficacy reached to a plateau with the increase of dose. The results suggest that low doses of rhubarb are useful for constipation curative and higher doses of the same substance are harmful from the overall point of metabolism view.

**Table 1:** Efficacy and adverse reactions value under different rhubarb dose

Value	Rhubarb 1	Rhubarb 2	Rhubarb 3	Rhubarb 4	Rhubarb 5	Rhubarb 6
Moderate positive reaction (efficacy)	12.32	24.68	50.27	49.46	49.18	50.07
Excessive positive reaction	0.00	0.00	− 5.19	− 10.42	− 16.33	− 32.94
Negative reaction	− 5.44	− 6.30	− 12.81	− 9.79	− 22.35	− 19.60
Unexpected adverse reaction	− 2.14	− 5.12	− 9.85	− 10.77	− 15.48	− 17.11

### Dose–response curve fitting

**Fig. 4:**
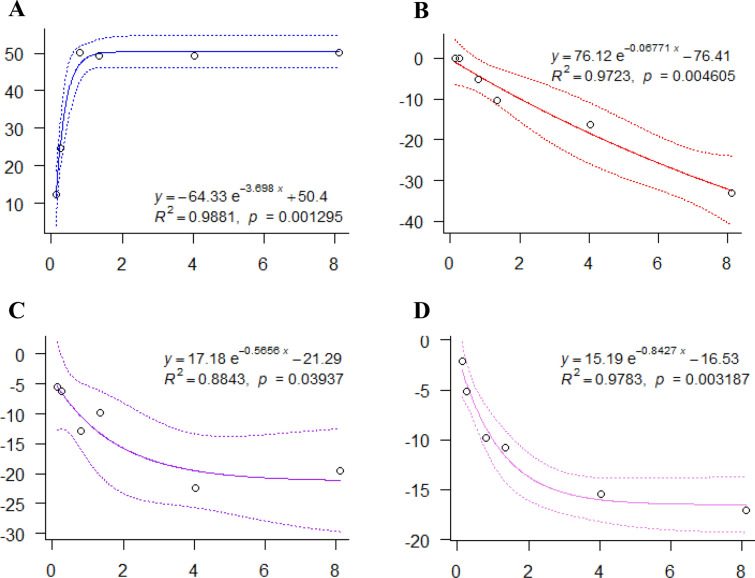
Dose–response relationships for rhubarb derived from metabolites. X-axis represents the dose examined in treatment group, and Y-axis represents the response. The dotted lines represent 95% CIs. (**A, B, C, D** represents curves of dose-efficacy, dose-excessive positive reaction, dose-negative reaction, dose-unexpected adverse reaction)

Dose–response models are regression models, in which the independent variable is called the dose, and the dependent variable is referred to the response (efficacy or adverse reactions). The dose–response relationship of rhubarb, as well as the formula of dose-efficacy, dose-excessive positive reaction, dose-negative reaction and dose-unexpected reaction are presented in Fig. 4.

It showed that the efficacy increased in the dose range of 0.135–0.81 g/kg and then reached to the plateau stage in the dose range of 0.81–8.1 g/kg, and the dose that provokes 90% response between the basal response and the maximal response—ED_90_ (90% effective dose) was determined as 0.69 g/kg, and ED_99_ (99% effective dose) was determined as 1.31 g/kg. Moreover, negative reaction and unexpected reaction showed similar trends, and they increased in the dose range of 0.135–4.05 g/kg and then reached to the plateau stage in the dose range of 4.05–8.1 g/kg, while dose-excessive positive reaction had different shape, which was on the upward trend in the dose range of 0.135–8.1 g/kg.

### Optimal dose selection

As one of the most effective laxative drugs, rhubarb is not only officially included in Chinese Pharmacopoeia, but also listed in European Pharmacopoeia and British Pharmacopoeia. However, it is applied in a wide dosage range. In this study, the established dose–response methodology based on metabolomics was employed to the optimal dose selection of rhubarb.

The goal of dose–response metabolomics study is to estimate an optimal dose based on the metabolic features. An optimal dose discovery rule by maximizing efficacy while limiting the adverse reaction is proposed. The acceptable adverse reaction dose that provokes 50% response between the basal response and the maximal response—TD_50_ (50% toxicity dose) for excessive positive reaction, negative reaction and unexpected reaction was calculated as 3.47 g/kg, 0.83 g/kg and 0.72 g/kg, respectively. In this study, the dose of 0.69 g/kg (corresponding to 7.66 g in clinic) was selected as the optimal dose because it was 90% of the effective dose and three adverse reactions were acceptable in this case.

**Fig. 5:**
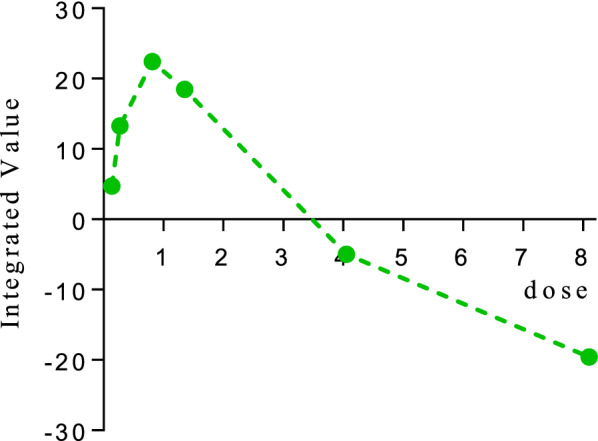
Dose–response relationships for rhubarb integrated by efficacy value and adverse reactions value

To further confirm the optimal dose, efficacy value and three types of adverse reactions value were integrated, the results showed that at the doses of 0.135, 0.27, 0.81 and 1.35 g/kg, rhubarb expressed varying degrees of effectiveness, with its best effect near 0.81 g/kg, while at the doses of 4.05 and 8.1 g/kg, the adverse reactions of rhubarb far outweighted the effect (Fig. 5). It was consistent with the above fitting result. In conclusion, our analysis showed a dose-dependent efficacy at around 1.50–9.00 g of rhubarb equivalents, beyond which there was no further increase in efficacy and a clear dose dependency in adverse effects; and the overall acceptability of treatment appeared to be optimal in the middle of the licensed range (3–15 g described in Chinese Pharmacopoeia). We therefore conclude that for the majority of patients receiving rhubarb, the lower range of the licensed dose will probably achieve the optimal balance between efficacy and acceptability. Clinical guidelines could incorporate these findings.

## Discussion

### Drug response: therapeutic effects and adverse reactions

**Fig. 6:**
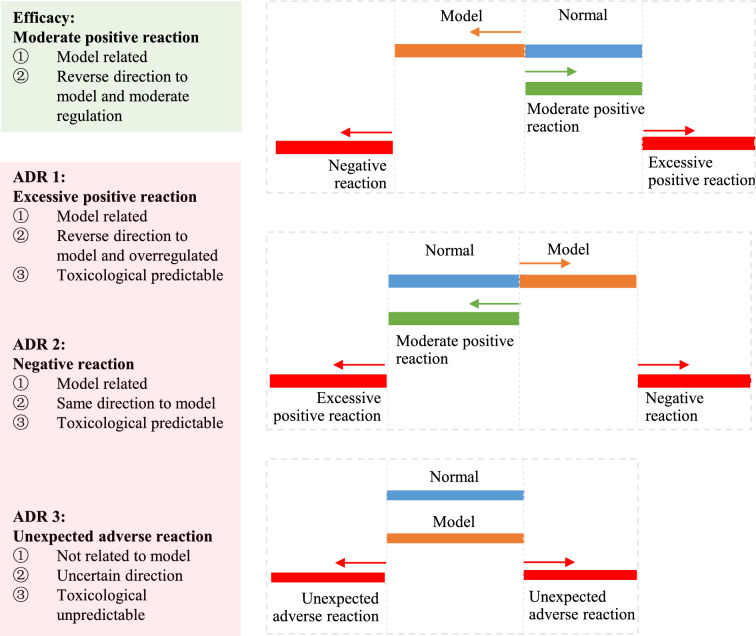
Characteristics of efficacy and adverse reactions in complex TCM-disease-individual system

There are two types of drug responses. Therapeutic effects are in line with the purpose of drug use, while adverse reactions are unintended and harmful responses to drug. The term “adverse reactions” encompasses all unwanted effects, which evokes no ambiguity [9]. Generally, adverse reactions are classified as type A or type B. Type A reactions are predictable from the known pharmacology of a drug, which may be caused by secondary pharmacology of a drug, representing actions that are different from the therapeutic effects. Type B reactions are idiosyncratic, bizarre or novel responses that cannot be predicted from the known pharmacology. Considering that not all adverse reactions fit into either category, additional categories including type C (continuing), type D (delayed use) and type E (end of use) have been developed [10, 11]. However, other criteria should be taken into account in a complex TCM-disease-individual system, we therefore propose a suitable classification system. Efficacy is defined as “moderate positive reaction”. Three types of adverse reactions are defined: “excessive positive reaction” is the disease-related response over-regulated by TCM, “negative reaction” is the disease-related response increased by TCM, and “unexpected reaction” is the unpredictable and novel response induced by TCM and independent from disease. The detailed characteristics of adverse reactions are summarized in Fig. 6.

### TCM dosage confirmation should attach importance to both therapeutic effects and adverse reactions

TCM dosage is of great significance in clinical application. In China’s oldest medical book “Prescriptions for fifty-two diseases”, the TCM dosage was recorded as a bunch of, a handful of or a few, which was mainly estimated. Through thousands of years, TCM dosage has gradually developed from fuzzy to accuracy, and the dosage range of TCM in Chinese Pharmacopoeia has been clearly defined. However, it is still a challenge to determine TCM dosage precisely for the following reasons: (1) TCM consists of dozens of ingredients, making it a very complex system; (2) the organism is dynamic and integrated, with different manifestations and symptoms in different stages of disease occurrence, making the disease a complex system; (3) there is a non-linear and complex interaction relationship between TCM and biological body. The role is reflected in the interaction between two complex systems of TCM and biological body, forming a higher-level system as a whole; (4) the response system includes not only therapeutic effects, but also adverse reactions, making it difficult to draw definite conclusions.

Safety and efficacy are two basic attributes of drugs. With the deepening understanding of the therapeutic effects and adverse reactions of TCM, dose–response relationship, that is the selection of dose for patients so as to maximize drug efficacy and minimize adverse reactions, is becoming a key issue of TCM modernization. Our idea is to obtain the accurate dosage of TCM application by integrating efficacy and adverse reactions summarized from metabolomics in complex TCM system to complex biological system.

### Integrated dose–response metabolomics with therapeutic effects and adverse reactions helps to scientifically and accurately characterize TCM dosage

The lack of objective and quantitative evaluation criteria makes it difficult to characterize the TCM action and provide the optimal dose with medication, which requires a rigorous approach. Up to now, the effective dose, minimum toxic dose and safety dose range of many TCMs are not completely clear. The toxicity classification of TCM is still based on the experience of the past dynasties, or the LD_50_ calculated from acute toxicity study. A set of scientific, objective and reasonable evaluation criteria for the efficacy and toxicity of TCM should be established.

The dose–response metabolomics strategy employed here can provide valuable information for the rational use of TCM. This approach is proposed based on a core concept that the whole metabolome is built from the physiological process of the body, and any metabolic reactions can be reflected. Dose is critical to the biological reactions, and the right dose can heal, while improper dose will inevitably lead to bias, including aggravating the disease, producing new lesion, or leading to adverse reactions. Whether it refers to the pathological changes caused by drug (poisoning) or the harmful rather than expected reaction of drugs, it can be distinguished and prescribed. In this study, the dose of 0.69 g/kg (corresponding to 7.66 g in clinic) was selected as the optimal dose. To illustrate the value of our approach, dose–response trends obtained from the integrated metabolomics features were compared to dose-dependent trends in traditional pharmacological data. In our previous research, the same six doses of rhubarb were performed on constipation model, and the dose–effect relationship was revealed by observing defection characteristics, intestinal propulsion rate, colonic content weight, etc., furthermore, the effective dose range (defined as EC_20_–EC_80_) was calculated as 0.67–5.37 g/kg (corresponding to 7.44–59.67 g in clinic) [5]. In this wide range of rhubarb, the increasing trend of the overall response was observed, however, efficacy and toxicity were indistinguishable. However, dose–response metabolomics employed in this study is helpful to reveal the efficacy and adverse reactions, thereby to confirm the optimal dose for application.

### Application of integrated dose–response metabolomics with therapeutic effect and adverse reactions

Integrated dose–response metabolomics is defined as “the prediction of optimal dose of a drug or xenobiotic intervention by integrating efficacy or adverse reactions derived from intervention metabolite signatures in individual based on a mathematical model”. By integrating metabolomics features representing therapeutic effect and adverse reactions, the optimal dose for TCM can be determined.

However, some possible limitations of the introduced approach should be addressed. First of all, the choice of a mathematical model varies in experiments, depending both on the shape of the graph and on the understanding of physiological processes. When we fit a model to data, best-fit values could be obtained for interpretation in the context. Our purpose is to find a model that describes the biological system as simple as possible. However, a complicated one may fit the data well and provide parameters that will help us understand the system and draw valid scientific conclusions. Secondly, in any synthetic evaluation system, it is a confusing to determine the weight of evaluation index. The method of objectively weighting each index should be further explored. What’ more, considering that it uses metabolites as a readout, dose–response metabolomics alone may not be able to deconvolute non-metabolic targets effectively. In these situations, integrating metabolomics with other omic technologies and biochemical assays may be helpful.

We also hope to point out the notable advantages of dose–response metabolomics. Firstly, metabolomics is sensitive to external stimulations and enables measurement of multiple small molecules, which reflects the actual physiological status of the biological system in real time. One of the aims in metabolomics is quantitation of metabolites in order to evaluate changes in response to diseases or treatment. Although absolute quantitative metabolomics is a powerful tool to obtain valuable metabolic information. Unfortunately, the absolute quantitative analysis of metabolic samples is often hampered by several reasons. It determines a class of metabolites that is often limited by the availability of commercial compounds, and focuses on some specific group of metabolites that is restricted to certain metabolic pathways [12]. Compared with absolute quantitative analysis, relative quantitative analysis is more economical and serviceable, either because this approach facilitates the comparison of metabolites changes response to stimulates, or because it provides further insight into the metabolic complexity and integrity. Secondly, the common challenge of metabolites identification faced in metabolomics studies does not exist in our approach. Identification of metabolites is the current bottle-neck and the comparison of its retention time, mass and fragmentation spectrum with those of authentic standards is still considered as the “gold standard”. Our practical approach ignored this time and cost consuming step, focusing on the changes of each metabolite in different groups, then it calculated the change degree as an overall efficacy or adverse reactions response. Thirdly, using a mathematical model can help us think about physiological processes, not only to comprehensively evaluate efficacy and adverse reactions, but also to obtain the optimal dose for clinical use.

## Conclusion

In this study, strategy of integrated dose–response metabolomics with therapeutic effects and adverse reactions is established for the first time, which is powerful to uncover the mysterious veil of TCM dosage. Methods for metabolomics data analysis in efficacy evaluation and adverse reactions assessment are detailed in both principle and practice. By integrating rapid, robust and reproducible pharmacological and toxicological information from metabolomics profiling, the optimal dose for TCM utility can be obtained. Our results show that integrated dose–response metabolomics with therapeutic effect and adverse reactions is an excellent method to reveal the TCM dosage, which is not only suitable for pre-clinical pharmacology and toxicity research, but also can be extended to clinical practice. It is envisaged that this strategy will provide the wealth of relevant data necessary to make critical personalized decisions for patient healthcare, and benefit the modernization and internationalization of TCM.

## Supplementary Information

**Additional file 1: **Quality Control of Rhubarb, **Figure S1** Multicomponent quantification of the rhubarb, Table S1 Calibration curve of 15 analytes. Animal Experiments, **Figure S2** Average food intake (A), weight (B), fecal pellet numbers (C) and fecal water content (D) of the normal group and model group, **Figure S3** Evacuation index (A) and fecal water content (B) of the normal group, model group and rhubarb groups, **Figure S4** Pathological sections of colons in the normal group, model groupand rhubarb groups. UPLC-Q-TOF/MS Data Acquisition, **Figure S5** Representative base peak intensity (BPI) chromatograms of serum samples from the normal group, model group and rhubarb groups in positive ion and negative ion modes, **Figure S6** PLS-DA score plots of serum samples classifying the normal group, model group and rhubarb groups and QC samples detected in positive (A1) and negative (A2) ion modes, Table S2 Mean values of 462 metabolic features for serum samples of the normal group, model group and rhubarb groups.

## Data Availability

The research data generated from this study are included in the article and additional files.

## Abbreviations

TCM: Traditional Chinese medicine
UPLC-Q-TOF/MS: Ultra-performance liquid chromatography tandem quadrupole time-of-flight mass spectrometry
RA: Relative abundance
AIC: Akaike’s information criterion
BIC: Bayesian information criterion
RSS: Residual sum of squares
ED: Effective dose
TD: Toxicity dose

## References

[1] Guo DA, Lu A, Liu L (2012). Modernization of traditional Chinese medicine. J Ethnopharmacol.

[2] Snyder R. Basic concepts of the dose–response relationship. In assessment and management of chemical risks. Amer Chem Soc. 1984;239:37–55.

[3] Yu XW, Gong QY, Hu KF, Mao WJ, Zhang WM (2017). Research on ratio of dosage of drugs in traditional Chinese prescriptions by data mining. Stud Health Technol Informat.

[4] Zhang CE, Niu M, Li RY, Feng WW, Ma X, Dong Q (2016). Untargeted metabolomics reveals dose–response characteristics for effect of rhubarb in a rat model of cholestasis. Front Pharmacol.

[5] Chen JQ, Chen YY, Tao HJ, Pu ZJ, Shi XQ, Zhang J (2020). An integrated metabolomics strategy to reveal dose-effect relationship and therapeutic mechanisms of different efficacy of rhubarb in constipation rats. J Pharm Biomed Anal.

[6] Yao CH, Wang L, Stancliffe E, Sindelar M, Cho K, Yin W (2020). Dose–response metabolomics to understand biochemical mechanisms and off-target drug effects with the TOXcms software. Anal Chem.

[7] Kaddurah-Daouk R, Kristal BS, Weinshilboum RM (2008). Metabolomics: A global biochemical approach to drug response and disease. Ann Rev Pharmacol Toxicol.

[8] Sindelar M, Patti GJ (2020). Chemical discovery in the era of metabolomics. J Am Chem Soc.

[9] Edwards IR, Aronson JK (2000). Adverse drug reactions: definitions, diagnosis, and management. Lancet.

[10] Aronson JK, Ferner RE (2003). Joining the DoTS: new approach to classifying adverse drug reactions. BMJ.

[11] Kaufman G (2016). Adverse drug reactions: classification, susceptibility and reporting. Nurs Stand.

[12] Xiao JF, Zhou B, Ressom HW (2012). Metabolite identification and quantitation in LC-MS/MS-based metabolomics. Trends Analyt Chem.

