# Supplementary material for: Integrated dose–response metabolomics with therapeutic effects and adverse reactions may demystify the dosage of traditional Chinese medicine
Source: Chin Med. 2022 Nov 19;17:130. doi: 10.1186/s13020-022-00687-4 (PMC9675273; doi:10.1186/s13020-022-00687-4)
Supplement: Supplementary file 1 — Additional file 1: Quality Control of Rhubarb, Figure S1 Multicomponent quantification of the rhubarb, Table S1 Calibration curve of 15 analytes. Animal Experiments, Figure S2 Average food intake (A), weight (B), fecal pellet numbers (C) and fecal water content (D) of the normal group and model group, Figure S3 Evacuation index (A) and fecal water content (B) of the normal group, model group and rhubarb groups, Figure S4 Pathological sections of colons in the normal group, model groupand rhubarb groups. UPLC-Q-TOF/MS Data Acquisition, Figure S5 Representative base peak intensity (BPI) chromatograms of serum samples from the normal group, model group and rhubarb groups in positive ion and negative ion modes, Figure S6 PLS-DA score plots of serum samples classifying the normal group, model group and rhubarb groups and QC samples detected in positive (A1) and negative (A2) ion modes, Table S2 Mean values of 462 metabolic features for serum samples of the normal group, model group and rhubarb groups. [file 13020_2022_687_MOESM1_ESM.doc]

**Supplementary Material**

**Quality Control of rhubarb.**

For preparation of rhubarb decoction, the rhubarb pieces were soaked in 10 times volume of water and boiled for 20 min, and the extraction was filtered with four layers of gauze; then 8 times volume of water was added into the residue and decocted for 15 min. The filtrates were combined and concentrated by rotary vacuum evaporator at 50°C.

*Standard substance*: Standards of gallic acid, catechin, aloe emodin 8-*O*-*β-D-*glucoside, rhein 8-*O*-*β-D*-glucoside, sennoside A, emodin 1-*O*-*β-D*- glucoside, chrysophanol 1-*O*-*β-D*-glucoside, chrysophanol 8-*O-β-D*-glucoside, emodin 8-*O*-*β-D-*glucoside, physcion 1-*O*-*β-D*-glucoside, aloe emodin, rhein, emodin, chrysophanol, physcion were purchased from Yuanye Ltd. (Shanghai, China) and the purity of all these compounds were higher than 95.0%.

*Chromatographic condition*: High performance liquid chromatography (HPLC) analysis was performed on an Acquity^TM^ Arc system (Waters, Milford, USA). Seperation was carried out on Sunfire C18 column (250mm × 4.6mm, 5 μm) maintained at 30 °C. The mobile phase was consisted of methanol (A) and 0.1% (v/v) formic acid water (B). The gradient elution was as follows: 0~3 min, 25% A; 3~8 min, 25%~28% A; 8~9 min, 28% A; 9~12 min, 28%~35% A; 12~26 min, 35% A; 26~35 min, 35%~39% A; 35~46 min, 39%~46% A; 46~60 min, 46%~74% A; 60~63 min, 74%~76% A; 63~72 min, 76%~80% A; 72~75 min, 80%~82% A; 75~85 min, 82% A; 85~88 min, 82%~25% A; 88~89 min, 25% A, at a flow rate of 1.0 mL⋅min^−1^. The signal was monitored at 260 nm. Figure S1A shows the chromatogram of the mixed standards. Figure S1B shows the representative HPLC chromatogram of the rhubarb decoction.


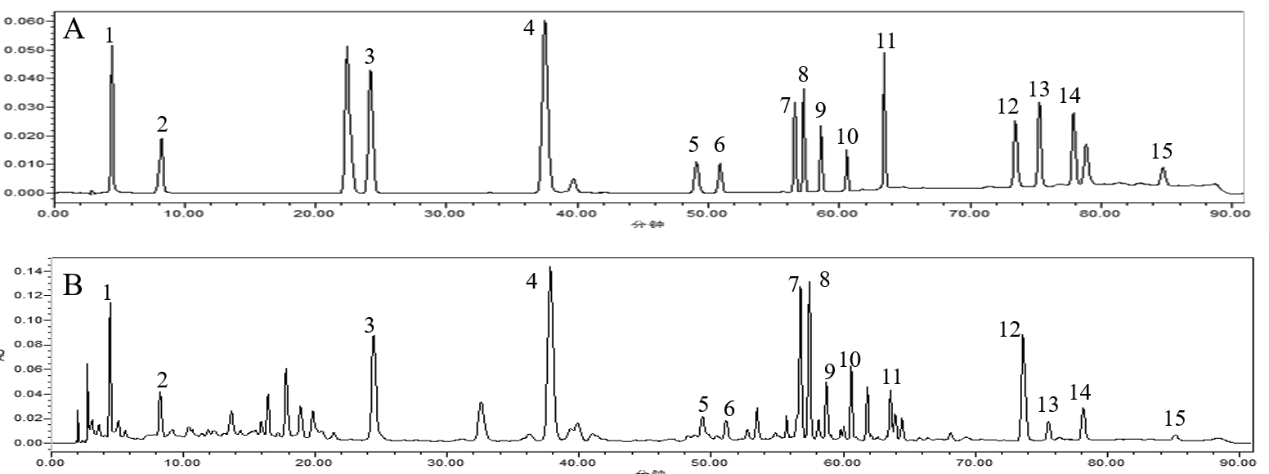


**Fig. S1 Multicomponent quantification of the rhubarb. (A) Chromatogram of the mixed standards, (B) HPLC profile of rhubarb extract, peak 1-15 represent gallic acid, catechin, aloe emodin 8-*O*-β-D-glucoside, rhein 8-*O*-β-D-glucoside, sennoside A, emodin 1-*O*-β-D- glucoside, chrysophanol 1-*O*-β-D-glucoside, chrysophanol 8-*O*-β-D-glucoside, emodin 8-*O*- β-D-glucoside, physcion 1-*O*-β-D-glucoside, aloe emodin, rhein, emodin, chrysophanol, physcion, respectively.**

*Preparation of standard solution and sample solution*: A mixed standard stock solution containing gallic acid (1, 0.023 mg/mL), catechin (2, 0.143 mg/mL), aloe emodin 8-*O*-β-D-glucoside (3, 0.043 mg/mL), rhein 8-*O*-β-D-glucoside (4, 0.176 mg/mL), sennoside A (5, 0.020 mg/mL), emodin 1-*O*-β-D-glucoside (6, 0.010 mg/mL), chrysophanol 1-*O*-β-D-glucoside (7, 0.010 mg/mL), chrysophanol 8-*O*-β-D-glucoside (8, 0.008 mg/mL), emodin 8-*O*-β-D-glucoside (9, 0.010 mg/mL), physcion 1-*O*-β-D-glucoside (10, 0.010 mg/mL), aloe emodin (11, 0.010 mg/mL), rhein (12, 0.011 mg/mL), emodin (13, 0.010mg/mL), chrysophanol (14, 0.008 mg/mL), and physcion (0.008mg/mL) was prepared in methanol. It was stored at 4℃ until use and filtered with 0.22 μm membrane prior to injection. The rhubarb decoction was diluted with methanol to 100 mg/mL and filtered with 0.22 μm membrane to obtain sample solution.

*Calibration curve*: The calibration curve for each constituent was established by plotting the peak area (y) versus the concentration (x) of each analyte. As shown in Table S1, all calibration curves showed good linearity (R^2^ > 0.9990) within the test ranges.

**Table S1** Calibration curve of 15 analytes

| Analytes | Regression equation | Correlation coefficient (*r^2^*) | Linear range  (μg/mL) |
| --- | --- | --- | --- |
| 1 | y = 2124426.22x - 7784.37 | 0.9994 | 0.023~0.480 |
| 2 | y = 213471.74x + 709.03 | 0.9999 | 0.143~3.000 |
| 3 | y = 1958407.68x + 1153.79 | 0.9998 | 0.043~0.900 |
| 4 | y = 847178.11x - 2608.24 | 0.9998 | 0.176~3.700 |
| 5 | y = 1048974.68x – 3251.38 | 0.9998 | 0.020~0.410 |
| 6 | y =1798861.24x -2107.11 | 0.9998 | 0.010~0.200 |
| 7 | y = 3480705.75x +1920.18 | 0.9998 | 0.010~0.200 |
| 8 | y =4910981.66x + 2099.75 | 0.9998 | 0.008~0.160 |
| 9 | y =2403198.56x +924.70 | 0.9998 | 0.010~0.200 |
| 10 | y =1369756.53x +774.21 | 0.9998 | 0.010~0.200 |
| 11 | y = 4423582.23x+10766.02 | 0.9997 | 0.010~0.200 |
| 12 | y = 3400095.57x -1832.72 | 0.9998 | 0.011~0.240 |
| 13 | y = 4527458.35x + 6810.37 | 0.9997 | 0.010~0.200 |
| 14 | y = 4978454.58x +6914.20 | 0.9997 | 0.008~0.160 |
| 15 | y = 1669182.09x – 6711.92 | 0.9998 | 0.008~0.160 |

*Precision*: The intra-day and inter-day precision for each constituent was investigated by determining the 15 analytes in six replicates during a single day and three consecutive days. Variations of the peak area were taken as the measures of intra-day and inter-day analysis precision to calculate the RSD. The intra-day variations of 15 analytes were 1.96%, 0.10%, 0.17%, 0.16%, 0.24%, 0.27%, 0.19%, 0.18%, 0.17%, 0.18%, 0.11%, 0.16%, 0.14%, 1.19%, 0.35%, and the inter-day variations of 15 analytes were 2.10%, 0.32%, 0.15%, 0.23%, 0.42%, 0.57%, 0.38%, 0.27%, 0.21%, 0.73%, 0.25%, 0.71%, 0.35%, 1.48%, 0.39%, which were less than 3.00%, suggesting good presicion.

*Stability*: Stability was investigated by analyzing the 15 analytes in sample solution at 0, 2, 4, 6, 8, 12, 24 h, respectively. Variations of the peak area were taken as the measures of stability to calculate the RSD. The RSD of 15 analytes were 2.33%, 2.03%, 2.79%, 1.77%, 2.14%, 2.08%, 1.50%, 2.00%, 1.46%, 1.77%, 1.92%, 1.62%, 1.69%, 2.92%, and 2.12%, respectively, which were less than 3.00%, suggesting the sample solution was stable with 24 h.

*Repeatability*: Repeatability was investigated by analyzing six rhubarb sample solution prepared in parallel. Variability was expressed in RSD. The RSD of 15 analytes were 2.56%, 2.17% 2.58%, 1.88%, 2.04%, 2.16%, 2.40%, 2.61%, 2.35%, 2.35%, 2.74%, 2.44%, 2.67%, 1.99%, 2.89%, which were less than 3.00%, suggesting good repeatability.

*Recovery*: Recovery was determined by using the method of standard addition within the same day. Mixed standard solution (100% of the known amount in sample) was spiked into rhubarb sample solution. The recovery results were calculated by comparing the difference between the spiked and the un-spiked sample that were analyzed under the same conditions. The recovery of 15 analytes were 90.57%, 102.00%, 102.21%, 98.48%, 92.26%, 90.72%, 97.81%, 104.80%, 103.67%, 104.95%, 111.07%, 95.91%, 116.53%, 117.38%, 87.43%, the RSD of 15 analytes were 0.85%, 0.02%, 0.01%, 0.06%, 1.19%, 0.37%, 4.40%, 0.48%, 0.06%, 0.07%, 1.70%, 0.53%, 1.22%, 2.03%, 2.89%, the overall recoveries lay between 87.43% and 117.38% with RSD less than 2.89 %, suggesting acceptable recovery.

*Multicomponents quantification of rhubarb:* Method validation results indicated that the established method was accurate for the determination of the 15 constituents in the rhubarb samples. Three rhubarb samples were prepared for quantitative analysis of 15 constituents. As a result, the concentrations of gallic acid, catechin, aloe emodin 8-*O*-β-D-glucoside, rhein 8-*O*-β-D-glucoside, sennoside A, emodin 1-*O*-β-D- glucoside, chrysophanol 1-*O*-β-D-glucoside, chrysophanol 8-*O*-β-D-glucoside, emodin 8-*O*-β-D-glucoside, physcion 1-*O*-β-D-glucoside, aloe emodin, rhein, emodin, chrysophanol, physcion were 1.462 ± 0.029, 8.285 ± 0.307, 3.277 ± 0.119, 15.586 ± 0.660, 0.987 ± 0.049, 0.528 ± 0.018, 1.491 ± 0.008, 1.065 ± 0.033, 0.746 ± 0.026, 1.423 ± 0.046, 0.333 ± 0.009, 1.690 ± 0.055, 0.194 ± 0.008, 0.304 ± 0.008, 0.213 ± 0.007 mg/g (mean ± SD, n = 3), respectively. The total content of aloe emodin, rhein, emodin, chrysophanol, and physcion was 0.273 %, which was in accordance with the quality standard of Chinese Pharmacopoeia (2020).

**Animal Experiments.**

*Animals:* Male Sprague Dawley rats weighing 50 ± 10 g (three-week-old) were purchased from Shanghai Slac Laboratory Animal Co. Ltd, China. Animal welfare and experimental procedures were in accordance with the guidelines of the Animal Ethics Committee of Shaanxi University of Chinese Medicine (No. 2020078). Animals were kept in a standard animal room with regulated temperature (20-24 °C), humidity (40-70 %), as well as a 12-h light/12-h dark cycle and were fed with food and water *ad libitum*. After one week of adaptive feeding, sixty-four rats weighing 100 ± 10 g were randomly divided into eight groups: the normal group, the model group, and six doses of rhubarb groups (n=8).

*Constipation model induction and evaluation:* The rats in the model group and rhubarb groups were given self-made HCD diet (white sugar: milk power: edible lard = 2:1:2, w/w/w) 0.2ml/10g twice a day for 7 days, while the rats in the normal group were given the same volume of normal saline by gavage. The general status was observed, food intake and weight of the rats were recorded. On the 8^th^ day, fresh stool pellets from individual rat were collected by metabolic cages during 12 h, their total numbers and weights were recorded. For determining the fecal water content, the stool pellets were dried until constant weight and their dry weight were measured. The fecal water content was calculated according to the following equation: Fecal water content (%) = (wet weight - dry weight) / wet weight × 100. The results showed that fur of the modeling rats became pallid and erect, and the activity decreased. Compared with the control group, the food intake decreased and the weight increased slowly in the model group. Fecal parameters were indication of the induction of constipation in the animals. Compared with the control group, HCD diet significantly reduced the fecal pellets number, fecal pellets weight and fecal water content, which means the constipation model was constructed.

**Fig. S2 Average food intake (A), weight (B), fecal pellet numbers (C) and fecal water content (D) of the normal group and model group. *P<0.05, compared with the normal group.**

*Dose design:* As the most commonly used herb for constipation, rhubarb has a strong curative effect, but may cause adverse reactions on liver, kidney and gastrointestinal system if used improperly. According to Chinese Pharmacopoeia, the daily human dosage of rhubarb is 3-15 g, which is converted into the equivalent dose of 0.27-1.35 g/kg for rats. Upon identifying the dose range that efficacy and adverse reactions could be presented, more wide doses around the Chinese Pharmacopoeia were tested. Therefore, six dosage groups were set up, which were 0.135 g/kg (Rhubarb 1), 0.27 g/kg (Rhubarb 2, equivalent to Pharmacopoeia low dose), 0.81 g/kg (Rhubarb 3, equivalent to Pharmacopoeia medium dose), 1.35 g/kg (Rhubarb 4, equivalent to Pharmacopoeia high dose), 4.05 g/kg (Rhubarb 5) and 8.1 g/kg (Rhubarb 6).

*Defecation characteristics of rhubarb:* After the constipation model was successfully reproduced, the diarrhea effect of rhubarb were observed. On the 9^th^ day, the rats in the model group and rhubarb groups were given the HCD diet once a day. Meanwhile, the rhubarb groups were orally given different doses of rhubarb decoction (0.1mL/10g) once a day for 3 days, additionally, the normal and model groups were give the same volume of normal saline. On the 12^th^ day, fresh feces from individual rat were collected by metabolic cages during 12 h, their total numbers, shapes (normal, semi-solid and liquid feces) and weights were recorded, and the evacuation index and fecal water content was calculated. The evacuation index was calculated according to the following equation: Evacuation index = numbers of normal feces × 1 + numbers of semi-solid feces × 2 + numbers of water liquid feces × 3. The results showed that the evacuation index increased in all groups received rhubarb, as well as the fecal water content. In addition, the high dose groups (Rhubarb 3- Rhubarb 6) showed the most significant changes.

**Fig. S3** **Evacuation index (A) and fecal water content (B) of the normal group, model group and rhubarb group. #P<0.05, compared with the normal group, *P<0.05, *P<0.01, *P<0.001, compared with the model group.**

*Pathological sections of colons:* On the final day of experiment, the rats were anesthetized with pentobarbital sodium and sacrificed for blood and tissue sampling. Histological tissue specimens of distal colon were fixed in 10% buffered formalin, dehydrated through graded alcohol and embedded in paraffin wax. Sections of 5 mm thickness were mounted on slides, stained with HE, and pathological changes were observed and photographed under an optical microscope. The normal group exhibited the morphologic characteristics of epithelial cells with well-demarcated crypt and goblet cells of colon tissues. In contrast, exfoliated intestinal epithelial tissues (black arrow) and inflammatory cell infiltration into mucosa (red arrow) were evident in constipation group. In groups of Rhubarb 2, Rhubarb 3 and Rhubarb 4, restoration of the colonic mucosa was increased compared with model group, which means the curing effect of rhubarb on constipation. While there existed exfoliated intestinal epithelial tissues (black arrow) and lymphocyte clusters and inflammatory infiltration (red arrows) in Rhubarb 5 and Rhubarb 6 groups.

**Fig S4 Pathological sections of colons in the normal group, model group and rhubarb group (HE-staining, 400×and scale bars=50 μm).**

*Sample collection for UPLC-Q-TOF/MS analysis:* On the final day of experiment, blood samples were collected by retro-orbital puncture and centrifuged at 3,000 rpm for 10 min, and serum samples were obtained and stored at -80°C until analysis.

**Data Acquisition.**

*UPLC-Q-TOF/MS analysis:* Ultra-performance liquid chromatography tandem quadrupole time-of-flight mass spectrometry (UPLC-Q-TOF/MS) was used for the metabolic profiling of rat serum. For serum sample preparation, methanol was added into the serum at the ratio of 3:1 to precipitate protein, and the mixture was vortexed for 1 min and centrifuged at 13,000 rpm for 10 min, then supernatant was obtained for UPLC-Q-TOF/MS analysis. UPLC-Q-TOF/MS analysis was performed by an Acquity^TM^ UPLC system (Waters, Milford, USA) coupled to a Synapt^TM^ Q-TOF mass spectrometer with electrospray ionization (ESI). Chromatographic separation was performed with an Acquity BEH C18 column (2.1 mm×100 mm, 1.7 μm) maintained at 35 °C. The mobile phase was composed of A (water containing 0.1% formic acid) and B (acetonitrile) under a gradient profile: 0-3 min, 95%-50% A; 3-7 min, 50%-40% A; 7-10 min, 40%-30% A; 10-14 min, 30%–5% A; 14-16.5 min, 5% B; 16.5-20 min, 5%-95% A. The flow rate was set to 0.4 mL/min^−1^ and the sample injection volume was 2 μL. Mass scan was set from m/z 100 to 1000 using both negative and positive electrospray ionization (ESI) modes. ESI source working parameters were as follows: ion source temperature of 120°C; desolvation gas temperature of 400°C; desolvation gas flow rate of 600 L/h; cone gas flow rate of 50 L/h; collision gas flow rate of 0.15 mL/min; capillary voltage of 3 kV for positive mode and 2.5 kV for negative mode, cone voltage of 30 V. In MS^E^ mode, two functions were performed, one at 6 eV and the other at 20–60 eV to induce fragmentation. All mass data were acquired using LockSpray™ to ensure mass accuracy and reproducibility. Leucine encephalin (m/z 556.2771 in ESI^+^, m/z 554.2615 in ESI^−^) was used as lock mass for exact mass measurement correction.

*Validation of UPLC-Q-TOF/MS method and metabolic profiles of serum samples:* At the beginning of the analytical batch, QC sample was continuously injected 5 times to balance the system. After the analysis of every eight samples, one injection of QC and corresponding solvent sample was analyzed throughout the workflow sequence. The reproducibility and reliability of the instrument status were assessed by the overlapped performance of QC spectral peaks. There were subtle changes in the overlaps of the total ion chromatogram (TIC) of each QC sample. But after preprocessing missing values by the 80 % rule, 69.43 % and 80.89 % of the variable peak intensity had relative standard deviation (RSD) less than 20 % severally among remaining positive and negative ions in the serum QC samples. These results were acceptable and collectively indicated that the method had excellent repeatability, stability and reliability for metabolic analysis. So the metabolism of each serum samples was profiled by the validated UPLC-Q-TOF/MS method. Representative base peak intensity (BPI) chromatograms of eight groups were illustrated in Fig S5 that showed good separation achieved within 20 min under the optimized conditions.

*Raw data processing:* Raw UPLC-Q-TOF/MS data were processed by MassLynx^TM^v4.1 software (Waters, Milford, MA) for peak detection, noise removal, filtering and alignment to generate a data matrix that was composed of retention time, m/z value and normalized ion intensity for each peak area. Major parameters were as follows: retention time range of 0.5-20 min, retention time window of 0.2, mass window of 0.05, peak width at 5% height of 1 s, markers intensity threshold of 25 counts, and noise elimination level of 6.

*Multivariate statistical analysis:* The processed data were imported into MetaboAnalyst. After the datasets were pareto-scaled, partial least squares-discriminant analysis (PLS-DA) was applied for supervised multivariate analysis to reduce the unnecessary information and reflect the clustering tendency of metabolic profile. As shown in Fig S6, the QC samples all clustered tightly close and the metabolic patterns of different groups were discriminated visibly with R^2^ and Q^2^ all over 0.8, indicating excellent fitness and predictive abilities of the constructed PLS-DA models. The model group was away from the control group, and to some extent, rhubarb-treated groups returned the metabolic profile of rats from disease to normal state and revealed an explicit dose-dependent trend.

**Fig S5 Representative base peak intensity (BPI) chromatograms of serum samples from the Control (A), Model (B), Rhubarb 1 (C), Rhubarb 2 (D), Rhubarb 3 (E), Rhubarb 4 (F), Rhubarb 5 (G) and Rhubarb 6 (H) in positive ion and negative ion modes.**


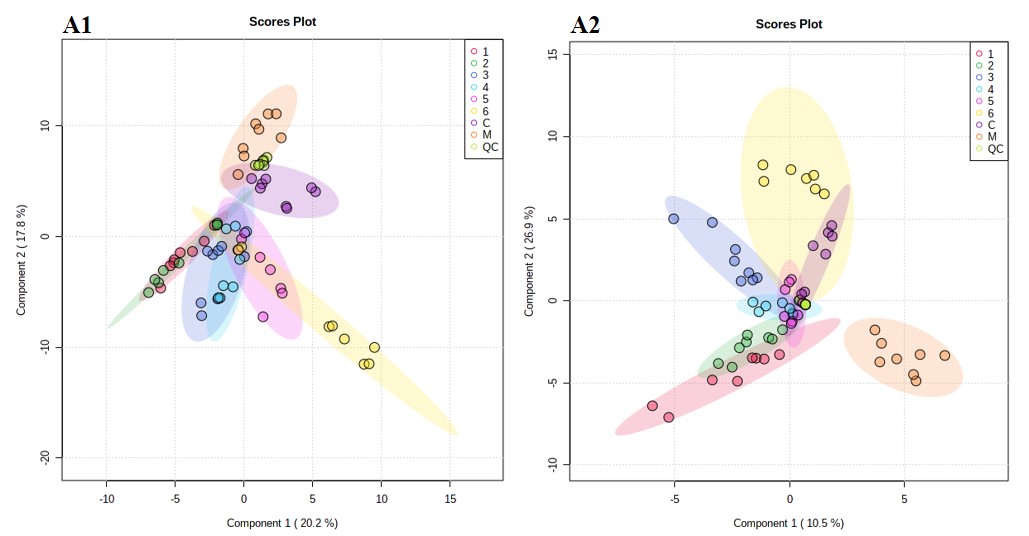


**Fig S6 PLS-DA score plots of serum samples classifying the Control, Model, six Rhubarb-treated groups and QC samples detected in positive (A1) and negative (A2) ion modes. Parameters of model were as follows: A1, R^2^ = 0.926, Q^2^ = 0.820; A2, R^2^ = 0.890, Q^2^ = 0.840.**

**Table S2 Mean values of 462 metabolic features for serum samples of 6 rhubarb groups, normal group and model group**

| **RT** | **ESI+** | **ESI-** | **normal group** | **model group** | **rhubarb 1 group** | **rhubarb 2 group** | **rhubarb 3 group** | **rhubarb 4 group** | **rhubarb 5 group** | **rhubarb 6 group** |
| --- | --- | --- | --- | --- | --- | --- | --- | --- | --- | --- |
| **0.83** | **131.932** |  | 0 | 9.8334925 | 11.66841875 | 19.711325 | 27.7352025 | 0 | 0 | 0 |
| **0.85** | **246.864** |  | 0 | 0 | 0 | 0 | 0 | 0 | 0 | 30.5884875 |
| **0.85** | **130.967** |  | 0 | 0 | 0 | 0 | 0 | 0 | 0 | 9.0232775 |
| **0.86** | **141.961** |  | 0 | 0 | 6.99850375 | 5.93423875 | 0 | 22.82785625 | 59.222825 | 66.2097625 |
| **0.87** | **113.966** |  | 19.9226925 | 0 | 0 | 9.94008125 | 0 | 0 | 70.6472875 | 73.1253625 |
| **0.88** | **217.861** |  | 0 | 0 | 0 | 0 | 0 | 0 | 29.05193625 | 38.97035 |
| **0.88** | **172.865** |  | 0 | 0 | 0 | 0 | 0 | 0 | 0 | 18.6042375 |
| **0.89** | **128.953** |  | 7.23993 | 1.238238625 | 3.426935 | 3.80556125 | 8.58541875 | 13.670975 | 23.15165 | 22.417275 |
| **0.89** | **112.959** |  | 0 | 0 | 0 | 0 | 0 | 0 | 0 | 11.953775 |
| **0.96** | **217.99** |  | 0.940935125 | 3.048202875 | 9.6322025 | 4.548979625 | 0 | 0 | 0 | 0 |
| **0.96** | **175.979** |  | 0 | 0 | 10.14308838 | 5.090878 | 0 | 0 | 0 | 0 |
| **1** | **158.964** |  | 0 | 0 | 0 | 0 | 0 | 0 | 0 | 11.96505 |
| **1** | **117.937** |  | 0 | 0 | 2.417494625 | 0 | 0 | 0 | 0 | 17.786475 |
| **1** | **100.935** |  | 0 | 0 | 0 | 0 | 0 | 0 | 0 | 13.7275625 |
| **1.02** | **145.932** |  | 0 | 0 | 0 | 0 | 0 | 0 | 0 | 16.8227875 |
| **1.04** | **217.861** |  | 0 | 0 | 0 | 0 | 0 | 0 | 0 | 28.9321875 |
| **1.06** | **113.966** |  | 0 | 0 | 0 | 0 | 0 | 0 | 53.11528125 | 64.345075 |
| **1.07** | **141.961** |  | 0 | 1.6935075 | 0 | 0 | 0 | 0 | 48.7909 | 55.6315875 |
| **1.08** | **112.96** |  | 0 | 0 | 0 | 0 | 0 | 0 | 0 | 10.590575 |
| **1.09** | **131.933** |  | 0 | 3.5113975 | 5.676975 | 8.089025 | 0 | 0 | 0 | 0 |
| **1.1** | **128.953** |  | 10.12811 | 1.598903 | 2.787695 | 3.88809125 | 11.41735625 | 12.42322375 | 23.865095 | 29.5428 |
| **1.25** | **217.106** |  | 3.8308045 | 12.01073188 | 13.7267175 | 18.8184 | 4.565086625 | 0 | 0 | 0 |
| **1.25** | **166.088** |  | 5.22007875 | 3.987185 | 5.07982125 | 5.3593475 | 0 | 0 | 1.253148625 | 1.965928 |
| **1.25** | **100.934** |  | 0 | 0 | 0 | 0 | 0 | 0 | 0 | 9.18083125 |
| **1.26** | **158.963** |  | 0 | 0 | 0 | 0 | 0 | 0 | 0 | 9.766305 |
| **1.26** | **145.932** |  | 0 | 0 | 0 | 0 | 0 | 0 | 0 | 15.0567875 |
| **1.26** | **141.961** |  | 0 | 0 | 0 | 0 | 0 | 0 | 0 | 35.1292125 |
| **1.26** | **128.954** |  | 0 | 0 | 0 | 0 | 0 | 0 | 17.0404625 | 17.1185775 |
| **1.26** | **117.938** |  | 0 | 0 | 0 | 0 | 0 | 0 | 0 | 11.5883625 |
| **1.4** | **261.132** |  | 6.8757025 | 27.96744375 | 24.9901375 | 28.602975 | 7.63558125 | 0 | 7.977805 | 5.16879125 |
| **1.53** | **305.158** |  | 5.174059125 | 23.7897575 | 21.7466125 | 23.3136 | 0 | 4.837285 | 6.4472175 | 0 |
| **1.65** | **349.184** |  | 4.53734375 | 15.67226375 | 18.3053475 | 20.9700875 | 5.44935 | 4.55019375 | 6.36479 | 5.85990625 |
| **1.75** | **393.21** |  | 0 | 7.87436875 | 9.2073075 | 8.60571 | 2.216479875 | 0 | 4.0334505 | 0 |
| **1.75** | **371.228** |  | 1.838096625 | 9.05731 | 10.73796625 | 8.5512525 | 0 | 2.35082625 | 3.86827875 | 4.42361625 |
| **1.77** | **130.161** |  | 0 | 1.247160625 | 1.6376475 | 2.885005 | 0 | 0 | 0 | 4.565106875 |
| **1.8** | **145.936** |  | 0 | 2.02625 | 0 | 5.3810025 | 0 | 0 | 0 | 0 |
| **1.81** | **147.934** |  | 0 | 2.406752125 | 0 | 6.52348125 | 0 | 0 | 0 | 0 |
| **1.83** | **437.236** |  | 2.414688125 | 10.276285 | 10.30035125 | 10.85296875 | 3.99233875 | 0 | 4.67933 | 4.027412875 |
| **1.9** | **476.308** |  | 2.026756375 | 3.5958715 | 0 | 4.3055195 | 0 | 0 | 0 | 0 |
| **1.92** | **582.292** |  | 0 | 0 | 0 | 7.952290125 | 0 | 0 | 0 | 0 |
| **1.93** | **632.816** |  | 0 | 0 | 0 | 0 | 0 | 0 | 0 | 5.521575125 |
| **1.99** | **834.889** |  | 0 | 0 | 3.850390625 | 0 | 0 | 0 | 0 | 0 |
| **2** | **405.984** |  | 0 | 5.697730875 | 10.02006625 | 0 | 0 | 0 | 0 | 0 |
| **2.16** |  | **195.813** | 0 | 9.97767875 | 0 | 0 | 0 | 0 | 0 | 0 |
| **2.36** | **396.801** |  | 0 | 6.95692875 | 6.6006025 | 0 | 0 | 0 | 0 | 0 |
| **2.62** |  | **195.813** | 0 | 14.0350325 | 0 | 0 | 0 | 0 | 0 | 0 |
| **2.89** |  | **197.809** | 0 | 29.368825 | 32.23985 | 0 | 0 | 0 | 0 | 0 |
| **2.89** |  | **195.812** | 0 | 0 | 22.1785375 | 0 | 0 | 0 | 0 | 0 |
| **3.06** | **430.296** |  | 0 | 0 | 4.15435375 | 0 | 0 | 0 | 0 | 0 |
| **3.1** | **254.142** |  | 17.2684 | 6.26142475 | 7.28373 | 16.534535 | 21.68713125 | 22.0878675 | 33.9901 | 31.2471 |
| **3.1** | **130.067** |  | 5.99846 | 2.679943 | 5.76619375 | 6.55420625 | 0 | 1.374881 | 0 | 0 |
| **3.13** |  | **329.161** | 0 | 23.39276738 | 21.6855875 | 18.9853725 | 15.23955038 | 10.5626525 | 10.62496625 | 0 |
| **3.16** |  | **462.286** | 12.47082875 | 32.39497125 | 29.131775 | 44.9151 | 51.3211125 | 20.099475 | 12.0060575 | 5.281441 |
| **3.28** | **337.199** |  | 3.71041125 | 0.41283175 | 1.105640625 | 1.87697575 | 3.884495625 | 4.88541875 | 9.5512075 | 11.70937 |
| **3.39** | **212.022** |  | 21.2222125 | 19.27443 | 26.02782875 | 20.7975875 | 8.523681 | 4.657080125 | 0 | 2.266610375 |
| **3.43** | **347.223** |  | 7.83754625 | 19.4513425 | 18.82975 | 20.201575 | 0 | 4.976460625 | 0 | 2.684160875 |
| **3.49** | **430.296** |  | 0 | 21.62008875 | 10.0328125 | 10.35582125 | 7.37092375 | 0 | 0 | 0 |
| **3.5** |  | **464.301** | 7.85335975 | 61.75053875 | 45.412275 | 71.4894625 | 50.966225 | 15.64243 | 16.396115 | 6.01786 |
| **3.57** | **163.077** |  | 4.81987375 | 0.7466565 | 1.77241 | 2.278885 | 4.426325 | 5.43289125 | 11.2079975 | 13.290125 |
| **3.57** | **105.035** |  | 0 | 0 | 0 | 2.056165 | 0 | 4.92957875 | 9.62343125 | 11.73656 |
| **3.59** | **261.112** |  | 0 | 0 | 3.026115 | 0 | 9.53760875 | 13.136405 | 26.08052 | 29.17972 |
| **3.59** |  | **197.809** | 0 | 9.120775 | 0 | 0 | 0 | 0 | 0 | 0 |
| **3.68** |  | **116.93** | 22.3663275 | 0 | 4.00173625 | 6.4728 | 12.80441625 | 0 | 0 | 30.4915 |
| **3.72** | **299.203** |  | 3.65312725 | 0 | 0 | 2.1644375 | 0 | 0 | 0 | 0 |
| **3.89** |  | **116.929** | 0 | 0 | 0 | 7.05615625 | 0 | 0 | 0 | 0 |
| **3.97** |  | **335.223** | 4.680654875 | 2.83472975 | 4.62831 | 17.97918438 | 24.72729 | 12.26503338 | 3.59371625 | 2.04070475 |
| **4.03** |  | **443.256** | 10.9358295 | 106.153 | 90.1719 | 14.90862025 | 0 | 12.84594163 | 9.745898875 | 0 |
| **4.04** | **454.354** |  | 0 | 7.788466125 | 7.499336375 | 0 | 0 | 0 | 0 | 0 |
| **4.04** | **373.274** |  | 20.86984 | 49.4512625 | 44.1802125 | 20.18165 | 21.91063125 | 14.79983688 | 0 | 0 |
| **4.04** | **355.263** |  | 49.251595 | 137.570125 | 102.9505875 | 49.45995 | 50.41051 | 34.80169625 | 10.149515 | 14.9748775 |
| **4.04** |  | **815.566** | 0 | 0 | 5.337485 | 11.40526775 | 0 | 0 | 0 | 0 |
| **4.04** |  | **407.28** | 60.7472525 | 81.27635 | 120.4644625 | 182.81644 | 232.1602 | 97.9966475 | 57.554925 | 45.16509475 |
| **4.05** |  | **116.93** | 0 | 0 | 0 | 0 | 0 | 0 | 0 | 25.342525 |
| **4.13** | **414.301** |  | 0 | 5.151231125 | 0 | 3.5593025 | 0 | 3.44239 | 1.0686095 | 0 |
| **4.14** | **357.279** |  | 15.336605 | 9.5088995 | 18.42729125 | 8.8679375 | 4.805190875 | 4.06957125 | 0 | 0 |
| **4.23** | **362.326** |  | 2.84722625 | 1.7091035 | 2.67553075 | 1.968825125 | 8.974385 | 4.68207 | 0 | 2.6592675 |
| **4.31** |  | **448.306** | 0 | 0 | 14.06217375 | 0 | 5.53602 | 0 | 0 | 0 |
| **4.49** | **299.201** |  | 37.301636 | 23.6657725 | 31.72593625 | 24.191775 | 15.55400875 | 11.23385375 | 5.660685 | 4.04297175 |
| **4.57** | **421.317** |  | 28.684175 | 49.020125 | 50.345925 | 56.3543875 | 30.9586 | 23.06039625 | 16.80683 | 18.8738625 |
| **4.57** | **376.26** |  | 36.7727625 | 62.7172375 | 64.2171 | 67.745675 | 37.223975 | 29.091975 | 22.4571375 | 20.88566375 |
| **4.6** |  | **395.243** | 0 | 7.75906375 | 8.44025375 | 0 | 0 | 0 | 0 | 0 |
| **5.04** | **460.271** |  | 0 | 4.24632375 | 3.4792315 | 4.31397625 | 5.3131075 | 0 | 3.36702 | 2.93880375 |
| **5.04** | **415.212** |  | 0 | 3.778505 | 3.5808575 | 4.4401675 | 5.10473375 | 5.4234375 | 0 | 3.8145925 |
| **5.11** | **301.216** |  | 23.41381375 | 13.09427375 | 16.88347125 | 20.078255 | 7.50684625 | 0 | 0 | 3.026483875 |
| **5.11** | **255.212** |  | 8.169197875 | 4.685547 | 6.41496875 | 6.8772575 | 0 | 0 | 0 | 0 |
| **5.4** | **357.279** |  | 7.9679675 | 18.03394125 | 26.732005 | 5.10787875 | 8.75509375 | 7.40494625 | 0 | 2.10855675 |
| **5.4** |  | **391.285** | 10.49824125 | 6.510980625 | 14.13802875 | 8.5478225 | 16.0142275 | 17.71118 | 6.424165 | 0 |
| **5.51** | **302.307** |  | 6.08461875 | 11.0241525 | 11.50709 | 9.669305 | 10.5950425 | 8.14552 | 8.298615 | 10.91835375 |
| **5.6** | **346.334** |  | 0 | 2.139265 | 2.6281875 | 2.29287 | 2.72758375 | 0 | 2.54764 | 0 |
| **5.66** | **468.31** |  | 16.69505 | 27.10616125 | 30.263525 | 33.0711875 | 16.142595 | 12.38514625 | 6.90225625 | 7.5291375 |
| **5.83** |  | **253.083** | 13.98197825 | 5.51448725 | 12.36921625 | 26.92472375 | 86.96434325 | 21.42866325 | 9.345479 | 0 |
| **6.19** | **494.325** |  | 28.6553 | 41.0263125 | 54.9631 | 53.4227875 | 30.6896375 | 23.1243375 | 11.320045 | 13.64749625 |
| **6.2** |  | **528.286** | 0 | 32.2029 | 41.960125 | 17.9954875 | 0 | 5.85595875 | 5.284272875 | 0 |
| **6.53** | **372.312** |  | 0 | 9.76677625 | 10.6048325 | 7.84154875 | 3.2173025 | 0 | 0 | 0 |
| **6.58** | **304.302** |  | 0 | 3.45802125 | 5.12348625 | 6.67430625 | 12.00013125 | 0 | 18.3097625 | 19.1372125 |
| **6.71** | **482.325** |  | 15.243345 | 12.2250375 | 15.0645625 | 15.35563875 | 8.4759425 | 7.84374625 | 2.74392875 | 0 |
| **6.84** | **568.341** |  | 56.1659625 | 114.77895 | 102.6498375 | 119.7797875 | 88.839575 | 63.8983375 | 27.8247 | 39.4362875 |
| **6.84** |  | **612.33** | 17.828592 | 23.2478 | 39.2959875 | 89.471825 | 103.18125 | 58.0795125 | 30.69505 | 15.49783625 |
| **6.84** |  | **602.301** | 5.916240875 | 76.700075 | 70.9464875 | 36.931245 | 4.0642165 | 17.45416375 | 11.70856375 | 0 |
| **6.86** |  | **544.268** | 0 | 0 | 24.58051875 | 19.0989 | 19.9605225 | 8.74032875 | 6.1986825 | 2.024507625 |
| **6.92** | **633.228** |  | 0 | 0 | 0 | 4.579939125 | 0 | 0 | 0 | 0 |
| **6.92** | **582.295** |  | 3.03170125 | 12.898035 | 12.1703275 | 10.82670875 | 0 | 0 | 0 | 0 |
| **6.92** | **566.326** |  | 0 | 4.93798875 | 5.68769875 | 6.74018375 | 0 | 0 | 0 | 0 |
| **6.92** | **544.341** |  | 246.489375 | 514.379375 | 510.840375 | 511.128875 | 336.291625 | 258.0665875 | 114.53525 | 162.779775 |
| **6.92** | **520.341** |  | 324.330625 | 431.361625 | 423.579875 | 429.9405 | 296.512875 | 228.2341875 | 107.454575 | 157.4885875 |
| **6.92** |  | **554.301** | 32.647955 | 184.256 | 167.9815 | 74.49874375 | 9.31033 | 49.19960875 | 37.10859625 | 0 |
| **6.92** |  | **528.309** | 28.793425 | 115.8568625 | 126.13525 | 103.8844625 | 74.0073 | 68.7378125 | 44.6693875 | 16.491684 |
| **6.92** |  | **504.309** | 37.74855 | 113.4563 | 116.9821625 | 93.3384375 | 76.7687625 | 69.3292 | 45.8480375 | 14.43841375 |
| **6.93** |  | **588.331** | 83.3787175 | 78.939575 | 138.78675 | 275.102375 | 323.626375 | 195.810875 | 119.790975 | 61.776405 |
| **6.93** |  | **578.301** | 27.32487 | 215.220875 | 208.583625 | 99.8944 | 10.401665 | 58.08042875 | 45.15391 | 4.6948175 |
| **6.93** |  | **564.33** | 83.623175 | 68.6437125 | 118.534675 | 221.3295125 | 304.227 | 186.268625 | 108.72205 | 54.1983725 |
| **7.41** | **570.357** |  | 6.61611875 | 13.739195 | 14.24652875 | 13.36401875 | 9.45389 | 0 | 0 | 0 |
| **7.41** | **184.075** |  | 6.54479375 | 14.85375 | 11.5211875 | 11.67715 | 8.52870125 | 0 | 4.00626 | 0 |
| **7.58** | **365.138** |  | 0 | 0 | 0 | 0 | 0 | 15.90153875 | 0 | 0 |
| **7.59** | **163.077** |  | 11.555365 | 0 | 3.51707 | 4.16146 | 0 | 0 | 19.4253375 | 25.4164875 |
| **7.59** | **105.035** |  | 8.37841625 | 0 | 2.3232875 | 3.69596625 | 7.5214625 | 0 | 0 | 21.1804875 |
| **7.63** | **330.338** |  | 0 | 9.409461 | 9.974524875 | 7.583612125 | 0 | 5.584101375 | 0 | 3.442252 |
| **7.77** | **105.035** |  | 9.85272625 | 0 | 0 | 3.594695 | 0 | 0 | 0 | 18.84195 |
| **7.78** | **163.077** |  | 13.1034875 | 0 | 3.8318375 | 4.64850125 | 0 | 0 | 0 | 25.4434375 |
| **7.8** |  | **452.278** | 12.38519625 | 62.3946875 | 59.66465 | 43.196025 | 35.53115 | 31.407925 | 23.1476625 | 8.3504625 |
| **7.89** | **991.674** |  | 0 | 174.7247625 | 113.4559375 | 78.523 | 24.7500125 | 3.80695875 | 0 | 0 |
| **7.91** | **771.966** |  | 0 | 3.86027375 | 0 | 4.307023625 | 0 | 0 | 0 | 0 |
| **7.91** | **604.239** |  | 0 | 6.02746625 | 0 | 8.26233075 | 0 | 0 | 0 | 0 |
| **7.91** | **602.239** |  | 4.284067125 | 7.073912125 | 7.07449825 | 8.565091375 | 0 | 0 | 0 | 0 |
| **7.91** | **534.298** |  | 18.699275 | 39.81739625 | 41.34370125 | 46.35154875 | 22.27798 | 19.4043 | 9.9048925 | 9.22733625 |
| **7.91** | **518.325** |  | 19.38315 | 15.6908875 | 17.8917375 | 23.224825 | 24.4074375 | 17.1941125 | 12.2499425 | 12.69483625 |
| **7.91** | **496.34** |  | 1315.7955 | 1936.275 | 1824.34875 | 1892.82 | 1508.43525 | 1107.789625 | 662.737125 | 747.899375 |
| **7.91** | **478.33** |  | 27.625375 | 35.30392875 | 40.0368875 | 42.20205 | 32.200025 | 24.543 | 14.91451875 | 17.14045 |
| **7.91** | **314.031** |  | 7.216415 | 2.6714225 | 4.60534125 | 5.04020375 | 7.62111625 | 4.954441625 | 4.523075125 | 8.60466375 |
| **7.92** | **595.263** |  | 5.361475 | 0 | 0 | 0 | 0 | 0 | 0 | 5.133595 |
| **7.92** | **585.229** |  | 8.12236375 | 2.24625125 | 0 | 8.70509875 | 8.9542475 | 9.39030625 | 6.45683875 | 5.75801625 |
| **7.92** | **524.307** |  | 9.45736 | 10.15229125 | 12.48096 | 13.88027 | 9.8862625 | 0 | 0 | 3.278156 |
| **7.92** |  | **480.309** | 193.326 | 530.84275 | 502.61725 | 412.431625 | 313.0955 | 340.22625 | 273.389 | 86.300875 |
| **7.93** | **578.259** |  | 0 | 0 | 0.677382375 | 0.9672845 | 5.143652875 | 0 | 0 | 0 |
| **7.93** | **184.075** |  | 0 | 9.47584625 | 0 | 0 | 0 | 3.72637875 | 0 | 0 |
| **7.93** |  | **612.26** | 30.8980375 | 28.6588125 | 31.6078125 | 44.3751875 | 53.89785 | 48.96165 | 41.72075 | 19.17160625 |
| **7.93** |  | **557.321** | 7.17162375 | 11.4516 | 0 | 11.2547875 | 12.25205125 | 16.537075 | 17.25565 | 5.55374125 |
| **7.93** |  | **540.33** | 374.096775 | 311.32425 | 474.2995 | 845.848 | 1078.958125 | 750.6725 | 540.267125 | 260.4918625 |
| **7.93** |  | **530.301** | 146.349375 | 651.8215 | 563.88375 | 263.7438375 | 27.2867375 | 201.9524 | 189.6877 | 24.13188375 |
| **8.09** | **303.233** |  | 24.897745 | 51.1259 | 50.19005 | 48.080275 | 34.1345375 | 15.66541 | 15.26641875 | 11.65140625 |
| **8.11** |  | **319.227** | 36.30000375 | 72.2764375 | 92.8123625 | 159.438425 | 183.9548875 | 79.104025 | 54.7488125 | 20.30622125 |
| **8.38** | **149.025** |  | 0 | 0 | 0 | 4.82947375 | 0 | 0 | 0 | 0 |
| **8.51** | **522.356** |  | 135.20625 | 324.91075 | 309.76025 | 278.299875 | 223.12475 | 166.329675 | 104.5005875 | 114.7691 |
| **8.51** |  | **566.346** | 52.76082375 | 66.930375 | 114.639425 | 203.1469125 | 253.852875 | 146.193025 | 93.70605 | 43.8454625 |
| **8.51** |  | **556.317** | 18.8481675 | 205.046375 | 194.74275 | 84.47529375 | 8.84314375 | 47.4126475 | 40.35609375 | 3.49919125 |
| **8.51** |  | **506.325** | 20.9263625 | 110.8895 | 120.165375 | 82.61105 | 59.2760125 | 53.6894 | 34.2802375 | 11.44478875 |
| **8.53** | **276.055** |  | 0 | 0 | 0 | 0 | 4.176519875 | 0 | 9.1573925 | 11.97781275 |
| **8.53** | **219.992** |  | 3.332218125 | 0 | 0 | 0 | 0 | 0 | 0 | 8.213015 |
| **8.53** | **149.025** |  | 220.0465 | 41.3670125 | 55.7341625 | 90.1514 | 204.4042 | 243.931125 | 343.396875 | 347.702875 |
| **8.53** | **121.031** |  | 26.2229625 | 5.271335 | 6.74413 | 11.01936375 | 25.4622 | 28.771325 | 39.797225 | 44.41105 |
| **8.8** | **522.356** |  | 0 | 0 | 12.44337875 | 0 | 0 | 0 | 0 | 0 |
| **8.81** | **400.342** |  | 10.32992625 | 32.827975 | 30.9031375 | 24.31499625 | 12.9875725 | 0 | 8.062885 | 10.22237 |
| **9.18** | **548.37** |  | 5.69134125 | 5.667388 | 5.1738675 | 6.64044125 | 5.8819675 | 0 | 0 | 3.2924075 |
| **9.23** | **510.358** |  | 24.143275 | 15.3991 | 14.9384375 | 16.5312375 | 13.8767375 | 12.0231525 | 7.252335 | 8.21967 |
| **9.26** | **426.358** |  | 8.0018275 | 29.96415 | 29.9455 | 20.77497875 | 0 | 0 | 9.25512 | 7.17421 |
| **10.03** | **524.372** |  | 0 | 149.118875 | 0 | 0 | 0 | 0 | 0 | 0 |
| **10.03** | **358.369** |  | 0 | 4.6515345 | 0 | 0 | 3.02283375 | 0 | 0 | 0 |
| **10.42** | **482.325** |  | 13.90839625 | 24.60505 | 19.24295 | 12.42124375 | 15.8064725 | 14.35107125 | 12.84627 | 15.62921375 |
| **10.44** |  | **480.31** | 15.97841375 | 69.888925 | 69.0112625 | 47.8612375 | 43.452725 | 41.09805 | 27.816875 | 12.99004625 |
| **10.56** | **613.257** |  | 2.439709875 | 0 | 0 | 3.101019375 | 0 | 0 | 0 | 0 |
| **10.56** | **562.329** |  | 12.56945625 | 33.0696925 | 28.06953 | 26.91768625 | 13.94413625 | 14.6796225 | 9.20831125 | 0 |
| **10.56** | **552.348** |  | 5.9204925 | 12.37120875 | 12.6508775 | 10.12519 | 0 | 0 | 0 | 0 |
| **10.56** | **524.372** |  | 924.904875 | 1399.814625 | 1241.59375 | 1082.82675 | 914.724875 | 877.482 | 575.33475 | 603.027125 |
| **10.56** | **506.362** |  | 14.4778375 | 29.6392625 | 18.4642625 | 17.875975 | 13.9691875 | 13.78437625 | 10.131155 | 10.152065 |
| **10.57** | **616.268** |  | 0 | 1.526964875 | 1.5500945 | 0 | 2.16339125 | 0 | 0 | 2.1004425 |
| **10.57** | **240.101** |  | 3.991172875 | 1.240811625 | 0 | 2.434153125 | 0 | 3.709119375 | 4.23423 | 4.831615 |
| **10.58** | **626.299** |  | 0 | 0 | 0 | 0 | 0 | 5.172824125 | 0 | 0 |
| **10.58** | **606.29** |  | 0 | 0 | 1.584477375 | 0 | 4.48466375 | 0 | 0 | 0 |
| **10.58** |  | **640.292** | 26.897925 | 33.3042875 | 38.576625 | 44.714025 | 48.8025625 | 50.2684875 | 41.5447625 | 20.41640375 |
| **10.58** |  | **568.362** | 247.5005875 | 199.936125 | 319.831625 | 511.047125 | 656.144125 | 532.89125 | 348.257375 | 199.7786375 |
| **10.58** |  | **558.333** | 111.7165075 | 503.18675 | 439.946375 | 207.8660625 | 21.159575 | 150.581775 | 129.5047875 | 21.63760375 |
| **10.58** |  | **508.34** | 92.7389875 | 254.391875 | 255.2095 | 188.315875 | 138.6503125 | 169.117375 | 124.423475 | 47.1539375 |
| **10.59** |  | **716.292** | 0 | 0 | 0 | 0 | 8.5424 | 0 | 3.610921625 | 0 |
| **10.98** | **428.374** |  | 3.13250125 | 16.2286975 | 12.39156125 | 8.00319 | 0 | 0 | 5.73212375 | 4.72992 |
| **11.24** | **510.397** |  | 0 | 0 | 2.7048775 | 1.69867125 | 2.45203125 | 0 | 0 | 0 |
| **11.56** | **398.229** |  | 0 | 0 | 0 | 0 | 3.42110875 | 0 | 5.8847875 | 0 |
| **11.85** | **538.388** |  | 0 | 2.86489375 | 3.37662625 | 0 | 2.5580625 | 0 | 0 | 2.07305125 |
| **11.89** | **256.264** |  | 10.86848375 | 18.01139375 | 14.4565975 | 10.2748825 | 7.42075125 | 0 | 7.834155 | 0 |
| **12.11** | **329.247** |  | 31.9723625 | 68.3362375 | 60.0537 | 49.6728375 | 35.7181625 | 28.1029375 | 22.00536 | 13.8432125 |
| **12.12** |  | **327.233** | 19.45329625 | 42.3322625 | 72.56965 | 83.036275 | 98.23925 | 62.2714125 | 32.8976625 | 14.8119125 |
| **12.16** | **321.199** |  | 0 | 16.5300625 | 14.78895725 | 0 | 0 | 0 | 0 | 0 |
| **12.16** | **255.212** |  | 9.0826725 | 22.5475675 | 22.79089875 | 19.2698 | 9.46735125 | 8.163705 | 3.242081625 | 3.302481125 |
| **12.27** |  | **339.226** | 0 | 14.05391625 | 17.1850625 | 0 | 0 | 0 | 0 | 0 |
| **12.3** | **141.115** |  | 0 | 0 | 0 | 0 | 4.6282875 | 0 | 0 | 0 |
| **12.34** | **282.28** |  | 29.829895 | 65.9073725 | 50.79995125 | 27.18017375 | 25.94254625 | 27.3817625 | 23.95422125 | 0 |
| **12.41** | **305.248** |  | 41.46675 | 65.193375 | 68.503825 | 58.7368375 | 43.6241875 | 35.6031125 | 23.132235 | 19.59398125 |
| **12.41** |  | **303.233** | 23.7077375 | 45.415425 | 78.2645875 | 86.6496375 | 107.779525 | 66.2471 | 43.21 | 17.2133625 |
| **12.64** | **337.17** |  | 5.334069625 | 0 | 0 | 2.31977875 | 4.538105625 | 4.0807875 | 3.07770375 | 0 |
| **12.65** |  | **333.229** | 0 | 3.54794125 | 12.77709375 | 2.7611935 | 0 | 2.51202525 | 3.600674125 | 0 |
| **12.66** | **331.264** |  | 7.65385375 | 9.8694575 | 10.0760625 | 7.06356 | 5.5619075 | 0 | 2.3633145 | 1.393297375 |
| **12.71** |  | **116.93** | 0 | 0 | 0 | 7.27472 | 0 | 0 | 0 | 0 |
| **13.12** | **209.158** |  | 0 | 1.426924125 | 1.481558875 | 1.56936075 | 0 | 0 | 0 | 3.92352 |
| **13.65** |  | **134.896** | 0 | 53.8822875 | 0 | 31.0907775 | 0 | 28.73543875 | 0 | 0 |
| **13.77** |  | **199.852** | 0 | 8.99569 | 0 | 15.123025 | 0 | 27.120575 | 0 | 0 |
| **13.78** | **147.935** |  | 0 | 0 | 7.41854 | 16.71313875 | 0 | 0 | 0 | 0 |
| **13.78** |  | **216.854** | 0 | 0 | 0 | 35.49165 | 0 | 0 | 93.7280125 | 138.767875 |
| **13.78** |  | **214.86** | 9.06986275 | 2.766933375 | 0 | 0 | 0 | 0 | 11.8742225 | 0 |
| **13.79** | **141.115** |  | 13.7120625 | 2.51665875 | 3.22962125 | 5.0080525 | 15.4863425 | 25.2400375 | 18.2697375 | 17.157125 |
| **13.82** | **284.299** |  | 10.233045 | 7.55579875 | 6.58303875 | 4.39028375 | 7.19621875 | 10.09024625 | 0 | 3.91501125 |
| **13.84** |  | **134.896** | 35.91903375 | 49.4783125 | 42.101425 | 28.87105125 | 6.1671775 | 0 | 26.92058 | 0 |
| **13.85** | **108.908** |  | 0 | 9.53150625 | 10.20767125 | 19.76461125 | 0 | 0 | 0 | 0 |
| **13.85** |  | **116.93** | 109.9543125 | 40.1276125 | 44.068225 | 54.6707375 | 67.2719125 | 92.235525 | 110.829425 | 128.41675 |
| **13.86** | **106.908** |  | 0 | 9.46251975 | 11.13973813 | 0 | 0 | 0 | 44.427575 | 31.18439375 |
| **13.87** | **149.934** |  | 0 | 7.415015375 | 0 | 0 | 0 | 0 | 0 | 0 |
| **13.9** | **310.311** |  | 0 | 6.19454375 | 5.610255 | 4.971752875 | 4.75626875 | 3.54855875 | 0 | 0 |
| **13.93** | **144.984** |  | 0 | 18.76359125 | 0 | 0 | 0 | 0 | 0 | 0 |
| **13.97** | **147.934** |  | 0 | 9.105142375 | 10.99932288 | 17.63791375 | 26.62758125 | 0 | 0 | 0 |
| **13.98** |  | **216.854** | 180.876825 | 46.58215 | 57.1020625 | 75.21605 | 120.1232625 | 130.9207625 | 155.151775 | 187.37175 |
| **13.98** |  | **199.852** | 63.3270625 | 19.3804725 | 21.5297125 | 27.4342 | 42.193875 | 47.7915125 | 57.7306875 | 64.103275 |
| **13.99** |  | **178.844** | 0 | 47.1338625 | 44.7426875 | 0 | 0 | 0 | 0 | 0 |
| **13.99** |  | **176.846** | 0 | 26.9401625 | 28.467625 | 0 | 0 | 0 | 0 | 0 |
| **14** |  | **214.86** | 22.5001325 | 4.86255625 | 6.42857125 | 8.890555 | 15.10427875 | 17.6325625 | 21.8110625 | 0 |
| **14.01** |  | **316.78** | 38.6233475 | 0 | 0 | 13.080735 | 27.89215 | 23.9517125 | 26.2085225 | 0 |
| **14.02** | **103.957** |  | 0 | 49.657625 | 64.0683275 | 0 | 0 | 0 | 0 | 0 |
| **14.02** |  | **125.93** | 14.47727875 | 2.77169375 | 2.79800125 | 0 | 10.93442125 | 0 | 11.38168125 | 18.174125 |
| **14.06** |  | **134.896** | 0 | 21.93285125 | 29.7471375 | 0 | 0 | 0 | 11.74803625 | 0 |
| **14.07** | **149.934** |  | 0 | 0 | 6.06064375 | 11.9617655 | 0 | 0 | 0 | 0 |
| **14.07** | **141.115** |  | 0 | 1.97855575 | 1.5390295 | 0 | 8.7788475 | 0 | 5.9255225 | 0 |
| **14.07** | **106.908** |  | 14.1963675 | 6.30666875 | 7.28786 | 11.61759875 | 0 | 0 | 0 | 0 |
| **14.07** |  | **116.93** | 112.8525125 | 33.15155 | 39.689725 | 41.38563375 | 70.5113375 | 78.30655 | 94.9264125 | 118.48325 |
| **14.08** | **108.908** |  | 11.24307125 | 4.700775 | 5.2285425 | 0 | 0 | 19.71399125 | 0 | 0 |
| **14.09** |  | **115.922** | 10.5990075 | 0 | 4.55043375 | 0 | 7.80793875 | 9.37486125 | 0 | 12.3172125 |
| **14.15** |  | **114.936** | 0 | 0 | 2.41167875 | 0 | 4.75242125 | 0 | 5.82729125 | 0 |
| **14.17** | **144.984** |  | 0 | 27.3275375 | 0 | 0 | 0 | 0 | 0 | 0 |
| **14.24** | **106.908** |  | 0 | 2.110029125 | 0 | 0 | 0 | 0 | 0 | 0 |
| **14.24** | **103.957** |  | 0 | 38.9033 | 0 | 0 | 0 | 0 | 0 | 0 |
| **14.24** |  | **199.852** | 47.079675 | 8.91188 | 14.93799125 | 0 | 23.89818 | 0 | 28.8327 | 52.9936125 |
| **14.25** |  | **216.854** | 108.53055 | 22.309575 | 29.87723125 | 0 | 66.3543875 | 42.6050625 | 66.1863875 | 109.829 |
| **14.25** |  | **214.861** | 15.734 | 0 | 3.82584125 | 0 | 10.08732125 | 0 | 11.05340375 | 0 |
| **14.25** |  | **178.844** | 0 | 21.7953125 | 35.1102625 | 0 | 0 | 0 | 0 | 0 |
| **14.25** |  | **176.846** | 0 | 23.66995 | 20.9054475 | 0 | 0 | 0 | 0 | 0 |
| **14.25** |  | **116.93** | 86.80702875 | 16.84287375 | 23.85511 | 21.179525 | 53.96085 | 37.7134625 | 48.60601 | 0 |
| **14.26** | **256.265** |  | 0 | 0 | 4.31589625 | 2.32669625 | 5.7579475 | 0 | 0 | 0 |
| **14.27** |  | **316.78** | 0 | 0 | 0 | 8.235635 | 23.5913125 | 14.3577625 | 18.73942 | 35.8223625 |
| **14.28** |  | **125.93** | 0 | 0 | 0 | 0 | 11.04939875 | 0 | 0 | 0 |
| **14.38** | **700.56** |  | 0 | 0 | 0 | 0 | 4.465545 | 7.18574625 | 5.693675 | 0 |
| **14.39** | **628.372** |  | 0 | 0 | 0 | 0 | 0 | 15.4770825 | 0 | 0 |
| **14.41** | **584.263** |  | 0 | 3.303005 | 0 | 0 | 0 | 0 | 0 | 0 |
| **14.43** |  | **178.844** | 0 | 0 | 11.10370125 | 0 | 0 | 0 | 0 | 0 |
| **14.54** | **524.45** |  | 0 | 0 | 0.688678 | 0 | 0 | 11.6775725 | 9.14282875 | 0 |
| **14.56** | **480.428** |  | 0 | 0 | 0 | 0 | 3.604638375 | 6.5818225 | 0 | 2.19616 |
| **14.58** | **463.4** |  | 0 | 0 | 0.37665075 | 0 | 0 | 7.108805 | 0 | 3.00546 |
| **14.88** | **149.025** |  | 0 | 0 | 0 | 0 | 28.6755825 | 45.8106 | 0 | 29.14715 |
| **15.12** | **338.342** |  | 149.903125 | 121.61145 | 113.862875 | 108.972925 | 114.828975 | 119.835265 | 128.5555 | 134.7305 |
| **15.12** | **303.306** |  | 0 | 4.886473375 | 4.27398875 | 4.31077875 | 4.65333625 | 4.814735 | 4.49760125 | 5.68533375 |
| **15.15** | **742.504** |  | 0 | 0 | 1.1257025 | 0 | 0 | 0 | 4.55660875 | 0 |
| **15.18** | **934.643** |  | 6.565371625 | 1.170063375 | 1.98971 | 3.05013375 | 0 | 0 | 12.399133 | 15.48989625 |
| **15.19** | **369.353** |  | 0 | 0 | 2.890762375 | 2.505154375 | 0 | 7.22293 | 0 | 5.68739 |
| **15.34** | **536.167** |  | 10.95888375 | 1.3308975 | 2.05664625 | 3.02995875 | 8.24801875 | 16.08742375 | 14.1829675 | 13.35857375 |
| **15.39** | **637.467** |  | 9.095875 | 0 | 0 | 1.6702275 | 9.34856 | 13.20369375 | 12.10437875 | 8.69376625 |
| **15.41** | **615.454** |  | 14.058775 | 0 | 1.37551275 | 0 | 0 | 16.16308375 | 14.10316625 | 9.04059125 |
| **15.42** | **593.442** |  | 14.29266375 | 0 | 0 | 1.769295 | 10.2368 | 16.9298925 | 14.054625 | 11.60611125 |
| **15.42** | **571.427** |  | 11.1768075 | 1.0120455 | 1.148605375 | 1.37754125 | 9.57376875 | 15.88268 | 13.46723875 | 6.6293875 |
| **15.45** | **549.417** |  | 0 | 0.8115725 | 0.628762375 | 0 | 0 | 0 | 10.889415 | 7.36444875 |
| **15.48** | **527.401** |  | 0 | 0 | 0 | 0 | 5.892544375 | 10.17875163 | 0 | 4.6005725 |
| **15.5** | **518.888** |  | 0 | 0 | 0 | 0 | 0 | 6.48314125 | 4.0032125 | 3.37979625 |
| **15.51** |  | **216.854** | 0 | 12.7788075 | 0 | 0 | 23.41649125 | 0 | 0 | 0 |
| **15.52** | **505.386** |  | 0 | 0.41959475 | 0 | 0 | 0 | 6.1732035 | 0 | 0 |
| **15.52** | **284.297** |  | 0 | 3.21894275 | 4.13369 | 2.890575 | 6.04637125 | 6.232275 | 0 | 0 |
| **15.55** |  | **316.78** | 29.51528625 | 7.08995 | 13.2852725 | 14.2756625 | 18.49710625 | 0 | 0 | 0 |
| **15.67** |  | **416.705** | 0 | 0 | 0 | 0 | 12.8088075 | 0 | 0 | 0 |
| **15.72** | **772.616** |  | 9.83416625 | 0 | 1.35568375 | 1.524325 | 6.9684225 | 12.16904875 | 11.32257375 | 6.98287 |
| **15.78** |  | **316.78** | 52.1826625 | 10.40884125 | 17.063275 | 17.58922375 | 32.304975 | 31.3318375 | 28.60016625 | 80.0247875 |
| **15.78** |  | **115.922** | 14.816175 | 8.34079 | 8.10838625 | 8.74389 | 10.58771 | 14.9760625 | 18.043225 | 0 |
| **15.8** |  | **216.854** | 94.7471625 | 29.1200375 | 36.199025 | 31.5937475 | 47.2577875 | 54.85695 | 52.38795 | 153.4024375 |
| **15.81** | **684.563** |  | 11.84507625 | 1.45889 | 1.780355 | 2.25564125 | 11.69935875 | 20.02496125 | 14.85587625 | 10.31589625 |
| **15.81** |  | **116.93** | 51.75545 | 15.31277625 | 16.1886125 | 24.39874 | 38.923225 | 30.5433875 | 36.7196 | 100.643925 |
| **15.83** | **640.537** |  | 18.8486825 | 2.1439125 | 1.88654125 | 2.82613375 | 12.88380375 | 26.2608725 | 20.19885625 | 12.5834875 |
| **15.83** | **610.185** |  | 0 | 1.508165 | 1.61118375 | 2.1850775 | 6.14865875 | 11.07858875 | 11.198035 | 8.6891425 |
| **15.94** | **491.43** |  | 11.59913 | 1.168759375 | 1.31895875 | 1.315055 | 7.746755 | 8.747915125 | 11.14419375 | 6.3593725 |
| **15.94** | **464.435** |  | 0 | 0 | 0 | 0 | 0 | 5.6499325 | 0 | 1.62319375 |
| **15.94** | **447.403** |  | 10.74806125 | 1.41180375 | 2.26157125 | 2.9089025 | 14.6838625 | 26.05944 | 20.12488875 | 14.298945 |
| **16.06** |  | **316.779** | 32.14700125 | 0 | 0 | 0 | 0 | 18.14505 | 0 | 43.467625 |
| **16.06** |  | **216.854** | 61.515575 | 0 | 0 | 24.81495 | 0 | 35.2464875 | 43.9233125 | 117.880675 |
| **16.06** |  | **116.93** | 41.407175 | 0 | 0 | 14.02225875 | 0 | 0 | 0 | 74.4214375 |
| **16.08** |  | **199.852** | 18.987175 | 0 | 0 | 10.30072625 | 0 | 13.7502375 | 0 | 42.0572125 |
| **16.3** |  | **216.854** | 0 | 0 | 0 | 0 | 0 | 0 | 0 | 110.06015 |
| **16.47** | **742.496** |  | 0 | 5.17778 | 7.03334875 | 6.47190875 | 0 | 9.01306375 | 11.1791 | 8.42733625 |
| **16.48** | **722.529** |  | 4.910895 | 1.48127875 | 1.87759875 | 2.73914375 | 4.4267105 | 4.280065 | 0 | 7.760735 |
| **16.48** | **663.455** |  | 93.4901625 | 15.99644 | 34.2755 | 44.61565625 | 94.8001625 | 120.036175 | 174.292 | 200.061875 |
| **16.49** | **708.512** |  | 0 | 8.27358375 | 15.7909125 | 0 | 35.4907875 | 37.026 | 0 | 0 |
| **16.49** |  | **216.854** | 0 | 0 | 0 | 22.1109125 | 0 | 0 | 0 | 136.4226375 |
| **16.52** | **736.544** |  | 6.878745 | 2.4569135 | 3.23250375 | 2.68954375 | 8.46697 | 0 | 12.0801325 | 9.71361125 |
| **16.53** | **817.576** |  | 6.99162875 | 1.8312215 | 2.676055 | 4.62126625 | 6.8570625 | 0 | 13.147325 | 14.41760125 |
| **16.53** | **701.411** |  | 0 | 2.618685125 | 3.998202125 | 0 | 0 | 0 | 11.0534725 | 0 |
| **16.53** |  | **199.852** | 0 | 0 | 0 | 8.585035 | 0 | 0 | 0 | 47.9121375 |
| **16.7** |  | **199.852** | 0 | 0 | 0 | 8.59331625 | 0 | 15.5282875 | 0 | 50.3735125 |
| **16.71** |  | **125.931** | 0 | 0 | 0 | 0 | 9.33886 | 0 | 0 | 0 |
| **16.72** |  | **214.86** | 0 | 0 | 0 | 3.6511775 | 0 | 0 | 0 | 18.4710375 |
| **16.79** |  | **316.779** | 30.50952375 | 0 | 2.50928 | 0 | 18.820225 | 0 | 0 | 58.9466875 |
| **16.8** |  | **216.854** | 87.52699375 | 15.3896075 | 15.3215675 | 23.8629275 | 55.8021 | 36.99525 | 0 | 162.63405 |
| **16.88** |  | **178.844** | 0 | 0 | 23.3048625 | 0 | 0 | 0 | 0 | 0 |
| **16.92** |  | **125.93** | 0 | 0 | 0 | 0 | 6.5509425 | 0 | 0 | 32.02257125 |
| **17.01** |  | **214.86** | 18.06631 | 0 | 0 | 0 | 8.13802375 | 0 | 0 | 23.797775 |
| **17.01** |  | **199.852** | 46.836195 | 0 | 0 | 9.75275125 | 29.7680775 | 14.57303375 | 0 | 73.8147125 |
| **17.01** |  | **115.922** | 21.2246475 | 0 | 4.4672375 | 4.39829 | 15.0130325 | 9.64108875 | 10.472425 | 27.1580425 |
| **17.03** |  | **116.929** | 122.821075 | 0 | 19.70802 | 0 | 61.153025 | 0 | 0 | 163.673 |
| **17.07** |  | **216.854** | 99.365825 | 0 | 15.27580125 | 17.5507925 | 51.1259375 | 0 | 26.983575 | 159.69185 |
| **17.08** |  | **316.779** | 0 | 0 | 0 | 7.40855 | 9.4691575 | 10.205105 | 14.95939375 | 37.0175225 |
| **17.09** |  | **134.896** | 14.8196475 | 0 | 0 | 5.82032 | 0 | 0 | 0 | 0 |
| **17.11** |  | **125.93** | 20.72848625 | 0 | 0 | 0 | 19.0610525 | 0 | 0 | 31.75995 |
| **17.12** |  | **178.844** | 0 | 0 | 27.89695 | 0 | 0 | 0 | 0 | 0 |
| **17.12** |  | **176.846** | 0 | 0 | 19.3302275 | 0 | 0 | 0 | 0 | 0 |
| **17.13** | **103.958** |  | 0 | 31.922925 | 0 | 0 | 0 | 0 | 0 | 0 |
| **17.14** | **144.984** |  | 0 | 14.47655 | 0 | 0 | 0 | 0 | 0 | 0 |
| **17.29** |  | **316.78** | 0 | 0 | 0 | 7.64102875 | 8.13219125 | 12.72133 | 0 | 0 |
| **17.29** |  | **199.852** | 0 | 0 | 11.08743375 | 10.466035 | 14.467855 | 0 | 0 | 49.73875 |
| **17.29** |  | **134.896** | 0 | 0 | 22.629825 | 9.7660125 | 5.6932425 | 0 | 0 | 0 |
| **17.31** | **144.984** |  | 0 | 23.29385 | 0 | 0 | 0 | 0 | 0 | 0 |
| **17.31** | **103.958** |  | 0 | 53.117325 | 0 | 0 | 0 | 0 | 0 | 0 |
| **17.34** | **149.934** |  | 0 | 0 | 0 | 5.00301875 | 0 | 0 | 0 | 0 |
| **17.36** | **106.908** |  | 0 | 4.028961375 | 0 | 6.89879625 | 0 | 11.01107 | 0 | 0 |
| **17.37** | **147.935** |  | 0 | 2.765568625 | 2.96574 | 7.22684375 | 0 | 0 | 16.79698375 | 14.0801275 |
| **17.39** |  | **178.844** | 0 | 49.430275 | 37.1051375 | 0 | 0 | 0 | 0 | 0 |
| **17.4** |  | **125.93** | 73.278825 | 20.0825 | 22.3478875 | 25.52756625 | 54.1597625 | 69.9435875 | 69.360875 | 83.6519625 |
| **17.41** |  | **176.846** | 0 | 33.22265 | 0 | 0 | 0 | 0 | 0 | 0 |
| **17.41** |  | **115.922** | 26.8660875 | 21.24086875 | 18.051175 | 26.341775 | 25.3958775 | 48.562325 | 69.4018075 | 58.591025 |
| **17.42** |  | **216.854** | 357.03275 | 185.6212 | 155.152125 | 262.4195 | 291.203375 | 453.498125 | 605.0165 | 545.696375 |
| **17.43** |  | **416.704** | 0 | 0 | 0 | 13.674495 | 0 | 0 | 33.595 | 0 |
| **17.43** |  | **316.779** | 97.5690375 | 0 | 0 | 69.4051 | 99.8801875 | 129.6275625 | 163.598 | 138.100375 |
| **17.43** |  | **214.86** | 45.348225 | 21.6447925 | 16.6749625 | 31.070775 | 35.6064375 | 55.1396125 | 71.51315 | 61.9591 |
| **17.43** |  | **199.852** | 125.763125 | 66.8533125 | 58.653375 | 95.40845 | 105.941575 | 160.2075375 | 213.77075 | 195.452 |
| **17.43** |  | **197.858** | 0 | 6.95648125 | 6.670425 | 10.13569125 | 12.8026025 | 19.399675 | 25.0127625 | 23.3978375 |
| **17.43** |  | **116.929** | 350.031375 | 157.1768375 | 132.282875 | 224.6725 | 241.47225 | 395.566125 | 548.267 | 441.670875 |
| **17.43** |  | **100.929** | 0 | 5.4083475 | 5.74358875 | 10.99889125 | 0 | 14.869025 | 19.6392125 | 15.2304925 |
| **17.44** |  | **538.61** | 0 | 4.95996125 | 4.221375 | 0 | 11.80284 | 18.264025 | 24.210875 | 21.269275 |
| **17.44** |  | **314.785** | 0 | 0 | 0 | 0 | 12.018625 | 0 | 25.5764125 | 0 |
| **17.45** | **112.898** |  | 0 | 0 | 6.284289 | 0 | 0 | 0 | 0 | 0 |
| **17.45** |  | **438.685** | 28.9416325 | 13.81532375 | 0 | 0 | 27.98655 | 46.4284625 | 76.689475 | 51.4851 |
| **17.45** |  | **165.936** | 13.70985375 | 6.23044 | 6.00842625 | 4.662945 | 9.01556875 | 8.80474875 | 11.1995325 | 0 |
| **17.46** |  | **700.51** | 0 | 0 | 0 | 4.53103375 | 3.5995115 | 9.71778875 | 14.87819625 | 8.03158 |
| **17.46** |  | **134.896** | 64.201575 | 73.87035 | 57.678825 | 48.9739875 | 14.2155 | 0 | 0 | 0 |
| **17.47** |  | **338.761** | 39.9608625 | 22.725465 | 16.8403 | 37.9758375 | 34.1746 | 67.2402875 | 88.7450125 | 61.4373125 |
| **17.47** |  | **222.842** | 0 | 0 | 1.3244775 | 3.0372375 | 2.5005825 | 0 | 7.82220125 | 0 |
| **17.5** |  | **256.847** | 13.29412 | 10.37854875 | 8.76356125 | 15.39748875 | 11.727365 | 29.2434875 | 40.19385 | 25.627325 |
| **17.53** |  | **146.967** | 0 | 3.800727625 | 0 | 5.8498775 | 0 | 0 | 19.673255 | 0 |
| **17.54** |  | **254.838** | 0 | 3.70371525 | 2.410845 | 3.49045 | 0 | 5.43960625 | 0 | 0 |
| **17.55** | **135.047** |  | 0 | 0 | 0 | 0 | 0 | 0 | 0 | 7.49033875 |
| **17.57** | **177.046** |  | 0 | 2.049972875 | 1.617168875 | 2.675183 | 0 | 0 | 0 | 2.79337375 |
| **17.58** | **365.105** |  | 0 | 0 | 2.433720125 | 3.8434675 | 0 | 7.83338125 | 0 | 8.651688625 |
| **17.59** | **194.119** |  | 40.70325 | 5.28157125 | 8.40925125 | 10.8352875 | 45.3816375 | 64.565825 | 56.4563125 | 65.866775 |
| **17.6** | **806.57** |  | 23.52443875 | 0 | 0 | 0 | 0 | 0 | 0 | 0 |
| **17.6** | **155.131** |  | 9.3012575 | 0 | 0 | 0 | 10.32718625 | 15.0367875 | 15.34191375 | 13.55975 |
| **17.6** | **126.105** |  | 10.83485 | 1.084510375 | 1.93279875 | 2.87237 | 9.152245 | 15.05301875 | 14.49360375 | 8.263205 |
| **17.61** | **222.114** |  | 0 | 0 | 2.65351125 | 0 | 8.56363 | 14.3172625 | 15.7083 | 14.33708625 |
| **17.61** | **141.115** |  | 244.6362625 | 51.0603875 | 66.1993875 | 106.3630375 | 282.0927 | 420.169375 | 426.502 | 371.494625 |
| **17.61** | **112.089** |  | 63.1295375 | 11.50943625 | 16.6707875 | 26.72055 | 74.1186375 | 106.6003 | 111.84645 | 97.1925875 |
| **17.61** |  | **102.958** | 0 | 0 | 0 | 0 | 0 | 0 | 10.10270875 | 0 |
| **17.65** | **112.898** |  | 0 | 0 | 5.979974375 | 0 | 0 | 0 | 0 | 0 |
| **17.66** |  | **134.896** | 0 | 44.4971125 | 0 | 0 | 0 | 0 | 0 | 0 |
| **17.67** | **804.551** |  | 0 | 0 | 0 | 0 | 5.98528625 | 0 | 0 | 0 |
| **17.69** | **265.964** |  | 0 | 0 | 0 | 0 | 0 | 4.024764125 | 0 | 4.07115125 |
| **17.69** | **226.953** |  | 7.586341875 | 0 | 0 | 0 | 0 | 0 | 0 | 7.306035 |
| **17.69** | **129.99** |  | 3.181816125 | 0 | 0 | 0 | 4.08355925 | 0 | 0 | 5.27117625 |
| **17.7** | **158.967** |  | 0 | 0 | 0 | 0 | 0 | 0 | 0 | 8.08861875 |
| **17.71** |  | **248.961** | 0 | 0 | 0 | 0 | 15.42218125 | 0 | 0 | 19.3275625 |
| **17.71** |  | **112.987** | 0 | 0 | 0 | 0 | 24.6146 | 0 | 0 | 31.1286875 |
| **17.72** |  | **316.949** | 0 | 0 | 0 | 0 | 11.38924625 | 0 | 0 | 13.0593625 |
| **17.81** |  | **197.809** | 0 | 44.4236125 | 39.24974875 | 0 | 0 | 0 | 0 | 0 |
| **17.81** |  | **195.813** | 0 | 42.2234875 | 0 | 0 | 0 | 0 | 0 | 0 |
| **17.82** |  | **160.844** | 0 | 23.0666 | 0 | 0 | 0 | 0 | 0 | 0 |
| **17.83** | **224.13** |  | 33.8855375 | 3.939825 | 4.718015 | 9.62321375 | 26.64945875 | 31.1067125 | 31.23615 | 32.4855 |
| **17.87** |  | **153.87** | 0 | 26.0241125 | 0 | 0 | 0 | 0 | 0 | 0 |
| **17.89** | **244.876** |  | 0 | 0 | 0 | 0 | 5.868075375 | 0 | 0 | 10.27425875 |
| **17.9** |  | **145.942** | 0 | 0 | 0.389299875 | 4.41406075 | 11.6700725 | 8.1122175 | 0 | 13.847855 |
| **17.91** | **185.871** |  | 0 | 0 | 0 | 0 | 4.220114375 | 7.428117875 | 0 | 7.525143875 |
| **17.93** | **478.331** |  | 0 | 0 | 0 | 0 | 3.76955375 | 0 | 2.586718375 | 0 |
| **17.93** | **130.936** |  | 7.8627985 | 3.5061075 | 3.425380375 | 6.70436325 | 6.049496125 | 9.2817475 | 13.7882775 | 0 |
| **17.94** |  | **189.933** | 11.8940535 | 0 | 0 | 4.330595 | 10.95603625 | 0 | 7.532468 | 14.6387025 |
| **17.94** |  | **101.953** | 10.3821415 | 0 | 0 | 3.921625125 | 9.17893875 | 0 | 6.98462875 | 12.04107 |
| **17.95** | **247.869** |  | 28.26704625 | 0 | 0 | 0 | 0 | 0 | 0 | 42.9182875 |
| **17.95** | **117.963** |  | 14.006495 | 4.51480625 | 0 | 0 | 0 | 0 | 0 | 0 |
| **17.95** |  | **294.71** | 0 | 11.7985425 | 0 | 0 | 0 | 0 | 0 | 0 |
| **17.96** | **174.959** |  | 0 | 5.94803875 | 0 | 9.82613325 | 0 | 0 | 0 | 0 |
| **17.97** | **167.156** |  | 0 | 3.98031275 | 0 | 4.84786975 | 0 | 0 | 0 | 0 |
| **17.97** |  | **164.841** | 0 | 87.8950875 | 59.5873125 | 0 | 0 | 0 | 0 | 0 |
| **17.98** | **190.861** |  | 8.407222125 | 0 | 0 | 1.55611025 | 0 | 0 | 0 | 12.076825 |
| **17.98** | **147.949** |  | 0 | 0 | 0 | 0 | 0 | 0 | 0 | 10.7511675 |
| **17.98** | **133.933** |  | 0 | 29.3517185 | 30.071805 | 57.12200625 | 0 | 0 | 0 | 0 |
| **17.98** |  | **337.87** | 0 | 0 | 0 | 4.01363175 | 9.6364175 | 0 | 0 | 12.348705 |
| **17.98** |  | **210.862** | 10.76053 | 0 | 0 | 0 | 9.22164875 | 0 | 0 | 10.294395 |
| **17.98** |  | **162.844** | 0 | 45.6639375 | 26.474925 | 0 | 0 | 0 | 0 | 0 |
| **17.99** | **324.794** |  | 16.99698275 | 0 | 0 | 0 | 0 | 0 | 0 | 24.04315 |
| **17.99** | **291.891** |  | 0 | 0 | 0 | 0 | 0 | 11.22232013 | 0 | 12.20270013 |
| **17.99** | **254.857** |  | 0 | 0 | 0 | 0 | 0 | 0 | 0 | 47.2483 |
| **17.99** | **250.865** |  | 99.1760165 | 0 | 0 | 0 | 86.97622075 | 122.5802331 | 155.31412 | 170.883125 |
| **17.99** | **224.866** |  | 21.675434 | 0 | 0 | 0 | 18.05346625 | 0 | 23.02269125 | 31.4978825 |
| **17.99** | **222.87** |  | 24.23514713 | 0 | 0 | 0 | 0 | 32.97314975 | 35.93631625 | 37.496975 |
| **17.99** | **205.868** |  | 0 | 0 | 0 | 0 | 0 | 0 | 8.497023125 | 9.09227525 |
| **17.99** | **180.877** |  | 0 | 0 | 0 | 0 | 0 | 0 | 0 | 9.9814 |
| **17.99** | **178.881** |  | 0 | 0 | 0 | 0 | 0 | 0 | 13.17672875 | 13.0606625 |
| **17.99** | **165.995** |  | 7.05290375 | 0 | 0 | 0 | 0 | 0 | 17.91507375 | 18.8795625 |
| **17.99** | **160.871** |  | 0 | 0 | 0 | 0 | 0 | 0 | 0 | 17.23225 |
| **17.99** | **143.962** |  | 56.7132375 | 4.49352875 | 10.11885625 | 13.664835 | 49.7023775 | 72.4441125 | 97.672925 | 92.066575 |
| **17.99** | **142.992** |  | 0 | 0 | 0 | 0 | 0 | 0 | 17.5547475 | 16.386625 |
| **17.99** | **138.981** |  | 0 | 0 | 0 | 0 | 9.06544 | 11.2550325 | 0 | 18.08125875 |
| **17.99** | **124.967** |  | 28.4293675 | 2.52468875 | 7.44661125 | 0 | 27.31263875 | 35.417525 | 63.74665 | 72.5249 |
| **17.99** | **119.96** |  | 0 | 1.20037625 | 1.7632625 | 2.781835 | 6.2274025 | 9.27463375 | 12.1698725 | 12.691175 |
| **17.99** | **103.96** |  | 9.7467025 | 0 | 0 | 0 | 0 | 0 | 0 | 15.0362 |
| **17.99** |  | **138.893** | 0 | 0 | 6.20760125 | 0 | 0 | 0 | 11.51757 | 7.571045 |
| **17.99** |  | **102.935** | 0 | 0 | 1.631424 | 0 | 31.058575 | 24.22703625 | 26.65252125 | 36.945875 |
| **18** | **474.72** |  | 0 | 0 | 0 | 0 | 5.709197 | 8.479795625 | 8.2124525 | 10.22958 |
| **18** | **400.791** |  | 9.02428725 | 0 | 0 | 0 | 0 | 0 | 0 | 13.4243625 |
| **18** | **398.796** |  | 0 | 0 | 0 | 0 | 7.283778625 | 10.481829 | 0 | 11.80265 |
| **18** | **372.796** |  | 0 | 0 | 0 | 0 | 0 | 0 | 0 | 17.332414 |
| **18** | **370.8** |  | 10.229992 | 0 | 0 | 0 | 9.760887125 | 0 | 0 | 15.17355 |
| **18** | **328.788** |  | 0 | 0 | 0 | 0 | 0 | 9.148375375 | 0 | 0 |
| **18** | **326.791** |  | 0 | 0 | 0 | 0 | 0 | 0 | 0 | 25.1262625 |
| **18** | **293.886** |  | 0 | 0 | 0 | 0 | 5.3370915 | 0 | 8.99062875 | 10.283075 |
| **18** | **226.865** |  | 0 | 0 | 0 | 0 | 0 | 11.47546625 | 12.65909375 | 13.240725 |
| **18** | **215.891** |  | 0 | 0 | 0 | 0 | 0 | 0 | 0 | 17.89075 |
| **18** | **188.865** |  | 0 | 0 | 0 | 0 | 0 | 0 | 0 | 16.620825 |
| **18** | **184.986** |  | 5.734985 | 0 | 0 | 0 | 6.53744 | 0 | 11.97758875 | 12.5440575 |
| **18** | **139.99** |  | 60.8271975 | 5.0859025 | 13.1630675 | 17.32991875 | 57.5949375 | 80.42375 | 112.190425 | 128.7648 |
| **18** | **133.976** |  | 0 | 0 | 0 | 0 | 7.30073625 | 0 | 14.0889275 | 15.4859875 |
| **18** | **115.966** |  | 68.958525 | 6.05386 | 14.60774625 | 19.65732 | 64.2839375 | 91.77345 | 138.5592375 | 149.4168625 |
| **18** | **100.96** |  | 59.7584 | 6.54760375 | 12.8093025 | 17.81344875 | 57.02125 | 76.5730125 | 109.0166625 | 115.443875 |
| **18** |  | **490.786** | 0 | 0 | 0 | 0 | 13.7914375 | 0 | 0 | 14.1984 |
| **18** |  | **488.789** | 11.57212975 | 0 | 0 | 0 | 11.7071825 | 0 | 0 | 14.10677 |
| **18** |  | **344.852** | 10.49188713 | 0 | 0 | 0 | 10.33632625 | 0 | 8.36844125 | 11.93936875 |
| **18** |  | **340.86** | 34.19810988 | 0 | 0 | 0 | 33.1269375 | 0 | 0 | 39.72484 |
| **18** |  | **296.87** | 12.447641 | 0 | 0 | 4.979760875 | 12.2686925 | 8.59235375 | 0 | 14.2445125 |
| **18** |  | **250.865** | 0 | 0 | 0.918978375 | 10.72167438 | 26.8476875 | 18.35807375 | 19.82921375 | 30.925075 |
| **18** |  | **208.828** | 0 | 7.2134525 | 8.2702225 | 0 | 0 | 0 | 0 | 0 |
| **18** |  | **206.875** | 0 | 0 | 0 | 0 | 33.08565 | 23.92624375 | 23.85947125 | 38.7151375 |
| **18** |  | **192.93** | 0 | 0 | 0 | 8.730880125 | 20.8131375 | 15.26555 | 0 | 24.93517 |
| **18** |  | **164.88** | 0 | 0 | 0 | 0 | 12.21406875 | 0 | 0 | 15.65515125 |
| **18** |  | **162.886** | 0 | 0 | 0 | 0 | 12.5892575 | 0 | 0 | 14.73581375 |
| **18.01** | **374.793** |  | 0 | 0 | 0 | 0 | 5.251353125 | 0 | 0 | 9.8212375 |
| **18.01** | **162.866** |  | 10.23202638 | 0 | 0 | 0 | 7.03462525 | 0 | 0 | 12.649126 |
| **18.01** |  | **330.831** | 0 | 0 | 0 | 0 | 3.083865 | 4.71585875 | 0 | 5.83711125 |
| **18.02** | **224.13** |  | 9.59478875 | 0 | 4.32400125 | 0 | 0 | 0 | 0 | 17.33683125 |
| **18.02** |  | **191.858** | 0 | 0 | 0 | 0 | 0 | 0 | 8.01301125 | 0 |
| **18.03** |  | **252.865** | 0 | 0 | 0 | 0 | 20.3613 | 0 | 0 | 20.2163125 |
| **18.06** | **232.89** |  | 0 | 0 | 0 | 0 | 0 | 6.516303625 | 0 | 0 |
| **18.18** |  | **153.87** | 0 | 0 | 23.101175 | 0 | 0 | 0 | 0 | 0 |
| **18.24** |  | **116.93** | 0 | 0 | 0 | 0 | 0 | 13.12173125 | 15.9267375 | 0 |
| **18.25** |  | **130.967** | 0 | 0 | 0 | 0 | 5.59706875 | 8.84913875 | 7.15659 | 12.35333 |
| **18.26** |  | **174.957** | 0 | 0 | 3.52191625 | 0 | 11.0134575 | 11.7253 | 19.140625 | 19.1962875 |
| **18.35** |  | **195.812** | 0 | 18.202075 | 0 | 0 | 0 | 0 | 0 | 0 |
| **18.46** |  | **174.957** | 0 | 0 | 0 | 0 | 14.4762625 | 12.40162875 | 15.16552625 | 23.99525 |
| **18.57** |  | **195.813** | 0 | 31.1687875 | 15.15148875 | 0 | 0 | 0 | 0 | 0 |
| **18.58** |  | **197.809** | 0 | 31.31535 | 17.92919375 | 0 | 0 | 0 | 0 | 0 |
| **18.59** |  | **160.844** | 0 | 20.6807625 | 11.398625 | 0 | 0 | 0 | 0 | 0 |
| **18.9** |  | **195.813** | 0 | 0 | 13.29709 | 0 | 0 | 0 | 0 | 0 |
| **19.14** |  | **195.813** | 0 | 0 | 17.51051375 | 0 | 0 | 0 | 0 | 0 |
| **19.35** |  | **197.81** | 0 | 0 | 34.73835 | 0 | 0 | 0 | 0 | 0 |
| **19.35** |  | **195.813** | 0 | 0 | 35.8088375 | 16.60592438 | 0 | 0 | 0 | 0 |
| **19.42** |  | **125.876** | 10.17279 | 0 | 2.89546625 | 4.133165 | 5.6693225 | 6.6715175 | 8.2167975 | 11.20387625 |
| **19.43** |  | **145.932** | 0 | 0 | 0 | 0 | 10.13831875 | 0 | 0 | 19.7003375 |
| **19.56** |  | **195.812** | 0 | 54.906425 | 39.68335 | 0 | 0 | 0 | 0 | 0 |
| **19.57** |  | **197.81** | 0 | 72.8820375 | 44.93655 | 0 | 0 | 0 | 0 | 0 |
| **19.58** |  | **201.804** | 0 | 10.36528875 | 0 | 0 | 0 | 0 | 0 | 0 |
| **19.59** | **158.964** |  | 0 | 0 | 0 | 0 | 0 | 0 | 0 | 11.6485 |
| **19.61** | **128.953** |  | 0 | 0 | 0 | 2.79396375 | 0 | 0 | 17.7929575 | 17.4796725 |
| **19.79** | **262.86** |  | 0 | 0 | 0 | 0 | 0 | 0 | 0 | 10.22514375 |
| **19.82** | **453.786** |  | 0 | 0 | 0 | 0 | 0 | 0 | 0 | 10.36426638 |
| **19.82** |  | **420.821** | 0 | 0 | 0 | 0 | 9.1695975 | 0 | 0 | 11.348885 |
| **19.85** | **128.953** |  | 0 | 0 | 0 | 0 | 4.604779125 | 0 | 0 | 9.60064 |
| **19.87** | **480.722** |  | 0 | 0 | 0 | 0 | 0 | 0 | 0 | 12.6766875 |
| **19.87** | **141.961** |  | 0 | 0 | 0 | 0 | 0 | 0 | 0 | 15.65415 |
| **19.88** | **111.974** |  | 0 | 0 | 0 | 0 | 0 | 0 | 0 | 16.47454625 |
